# Optimal Control Drives Ultrafast and Energy‐Efficient Magnetization Switching in Van der Waals Magnets

**DOI:** 10.1002/adma.202523059

**Published:** 2026-07-14

**Authors:** Mohammad H. Badarneh, PeiYu Cai, Elton J. G. Santos

**Affiliations:** ^1^ Institute for Condensed Matter and Complex Systems, School of Physics and Astronomy The University of Edinburgh Edinburgh UK; ^2^ Higgs Centre for Theoretical Physics The University of Edinburgh Edinburgh UK; ^3^ Donostia International Physics Center (DIPC) Donostia‐San Sebastián Spain

**Keywords:** device engineering, energy‐efficient switching, ultrafast magnetisation dynamics

## Abstract

The accelerating expansion of data‐centric technologies is sharply increasing the energy burden of information storage, placing unprecedented pressure on the efficiency of magnetic switching. Conventional field‐driven reversal, once the foundation of magnetic memory, has become impractical in modern architectures due to its high energy cost, limited spatial selectivity, and poor scalability, leading to the dominance of current‐driven techniques based on spin‐transfer‐ and spin–orbit‐torque (STT, SOT). Here we show that optimal control theory (OCT) enables precisely shaped magnetic‐field pulses that unlock ultrafast, deterministic, and exceptionally energy‐efficient magnetization switching in van der Waals magnets, making field‐driven reversal competitive with, and in some regimes superior to, established current‐based approaches. Using ${\mathrm{Fe}}_{3}{\mathrm{GaTe}}_{2}$, ${\mathrm{Fe}}_{3}{\mathrm{GeTe}}_{2}$, and CrSBr as representative systems, we show that OCT‐induced uniform spin rotations completed within the picosecond range (1−10 ps) with switching energies (9.7−0.94 nJ) up to two orders of magnitude lower than conventional field protocols (91.2−42.8 nJ). Optimized fields operate at amplitudes more than tenfold smaller than standard methods, and by exploiting material‐specific parameters such as damping and anisotropy, low femtojoule switching energies become attainable, surpassing SOT or STT schemes. Our findings suggest that, rather than being obsolete, OCT‐field implementations could complement or hybridize with current‐ and light‐driven approaches, opening a new design space where magnetic switching is tailored by the interplay of fields, currents, and photons to achieve unprecedented efficiency, speed, and scalability.

## Graphical abstract

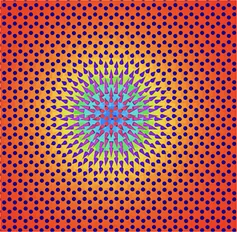

## 1 Introduction

The ability to reversibly control magnetization with minimal energy cost lies at the heart of condensed‐matter physics and is central to the development of sustainable information technologies [1, 2, 3]. In magnetic memory devices, writing data requires selective magnetization reversal [4, 5, 6, 7, 8], with every bit operation incurring a fundamental energy penalty. As artificial intelligence and advanced computing platforms proliferate, reducing the energy required per switching event has become a critical challenge. Meeting this demand requires new strategies that optimize magnetization dynamics while suppressing energy dissipation. Optimal control theory (OCT) provides a powerful framework to address this challenge. Rather than relying on trial‐and‐error scanning of possible control protocols, OCT identifies the trajectory that minimizes control energy, and hence Joule heating, while ensuring reliable switching [9, 10, 11]. OCT has been shown to be transformative in engineering, economics, biology, and physics [9, 10, 11, 12, 13, 14, 15, 16]. Nevertheless, its application in magnetism remains limited, leaving open the question of whether real materials and device‐relevant setups can benefit from its principles.

In the absence of such approaches, magnetization switching has been pursued through alternative mechanisms, including ultrafast optical pulses [17, 18, 19], spin‐polarized currents [20, 21, 22, 23, 24], microwave‐assisted reversal [25, 26, 27], and thermal gradients [28, 29]. Each comes with inherent trade‐offs: applying antiparallel fields above the coercive threshold results in slow, damping‐limited switching [30, 31], while reducing magnetic anisotropy lowers energy barriers at the expense of thermal stability [32, 33]. Precessional switching can be faster, but is notoriously prone to ringing instabilities and back‐switching if pulses are not precisely shaped [4, 34, 35, 36]. These limitations underscore the need for a more structured, predictive methodology to identify energy‐efficient switching protocols.

This challenge is particularly timely for emerging atomically thin van der Waals (vdW) magnets. These materials combine the use of earth‐abundant elements with facile external tunability and the stabilization of topological spin textures, offering promising opportunities for spintronics applications [37, 38, 39, 40, 41, 42, 43, 44, 45, 46, 47]. Recent advances in techniques have demonstrated a reliable control of the magnetization through current‐induced effects, such as spin‐orbit torque (SOT) in ferromagnet/heavy‐metal heterostructures [48] and intrinsic self‐SOT in ${\mathrm{Fe}}_{3}{\mathrm{GeTe}}_{2}\mathrm{magnets}$ [49]. These developments have enabled field‐free switching in both all‐vdW and hybrid heterostructures [50, 51], and have recently been extended to antiferromagnetic systems exhibiting intrinsic self‐torque and field‐free switching [52]. Comprehensive overviews of these advances can be found in recent reviews [53, 54]. In terms of materials, intensive research has been conducted in their exploration. Representative compounds such as CrSBr exhibit remarkable air stability, semiconducting behavior, and robust magnetization down to the monolayer limit [55, 56, 57, 58]. ${\mathrm{Fe}}_{3}{\mathrm{GaTe}}_{2}$ maintains room‐temperature (RT) ferromagnetism [59, 60], while ${\mathrm{Fe}}_{3}{\mathrm{GeTe}}_{2}$ can be chemically engineered to exceed its native Curie temperature of 220 K [61, 62]. These materials offer promising building blocks for spintronics devices, but it remains unknown whether their magnetization can be steered through OCT protocols and to what extent such control can deliver the energy reductions urgently needed for sustainable computation.

Here, we investigate magnetization switching in ${\mathrm{Fe}}_{3}{\mathrm{GaTe}}_{2}$, ${\mathrm{Fe}}_{3}{\mathrm{GeTe}}_{2}$, and CrSBr using optimally shaped magnetic fields. We show that OCT enables highly efficient reversal mechanisms that markedly outperform conventional approaches. In all three magnets, switching proceeds through coherent spin rotation on ultrafast timescales, with an optimal control path (OCP) that reduces both the field amplitude and Joule heating. For instance, CrSBr requires a peak field of 1 T for a switching time (τ) of τ=126 ps under conventional conditions, whereas the OCT protocol achieves the same switching time with a field of an order of magnitude smaller (0.11 T). More generally, vdW magnets with low damping values (α=0001–0.05) achieve switching in the femtojoule regime (∼6–12 fJ for τ=5–10 ns), positioning OCT as competitive with state‐of‐art current‐based methods used in leading technologies, such as spin‐transfer‐ and spin‐orbit‐torque magnetoresistive random access memory (STT‐MRAM, SOT‐MRAM), and voltage‐gate‐assisted spin‐orbit‐torque (VGSOT). Beyond uniform rotation, OCT also enables control over more complex magnetic textures. We demonstrate the reversal of a skyrmion state into a ferromagnetic (FM) configuration, where the OCP triggers the emission of concentric spin‐wave ripples that propagate across the system. Remarkably, this process achieves switching energies as low as 8.8 fJ within τ=4.7 ns. Such relaxation pathways are absent in minimum‐energy path (MEP) descriptions, underscoring the ability of OCT to uncover alternative, ultrafast switching mechanisms that expand the design space for low‐power spintronics devices.

## 2 Results

### 2.1 Optimal External Magnetic Fields

We first examine the magnetization reversal mechanism in monolayer vdW magnets subjected to optimally tailored external magnetic fields. Figure 1a–c depicts the reversal process in three systems (${\mathrm{Fe}}_{3}{\mathrm{GaTe}}_{2}$, ${\mathrm{Fe}}_{3}{\mathrm{GeTe}}_{2}$, and CrSBr) whose stable FM ground‐states are defined by their intrinsic anisotropies [55, 59, 63]. In ${\mathrm{Fe}}_{3}{\mathrm{GaTe}}_{2}$ and ${\mathrm{Fe}}_{3}{\mathrm{GeTe}}_{2}$ the spins align along ±z, while in CrSBr they align along ±y. In all cases, the switch proceeds through a coherent rotation of the spin ensemble (Movies S1–S3): magnetic moments rotate counterclockwise in the first half of the trajectory, reach the equatorial plane at t=τ/2, and subsequently reverse direction. Although nonuniform dynamics can emerge on longer time scales [64], coherent rotation remains a widely observed and technologically relevant reversal pathway, particularly at the nanoscale [65]. Ensuring stability of this mode has been central to industrial development of magnetic switching [4], given its robustness and low energy cost. The optimal control pulses that drive this process (green curves in Figure 1a–c) reveal distinct fingerprints of material‐specific properties such as damping, anisotropy, saturation magnetization, and the number of magnetic ions per unit cell. For instance, ${\mathrm{Fe}}_{3}{\mathrm{GeTe}}_{2}$ exhibits a pronounced precessional motion around its easy axis, in contrast to ${\mathrm{Fe}}_{3}{\mathrm{GaTe}}_{2}$ and CrSBr. Extraction of the corresponding optimal switching fields $B_{\mathrm{opt}}$ from the OCPs via Equation (5) show that ${\mathrm{Fe}}_{3}{\mathrm{GeTe}}_{2}$ requires the largest optimum field amplitudes, $B_{\mathrm{opt}}^{x},B_{\mathrm{opt}}^{y}∼5.2$ T, with strong oscillations (Figures 1e). This behavior reflects the compound's large magnetic anisotropy and relatively low damping, underscoring how intrinsic material parameters dictate the efficiency and character of optimal field‐driven switching.

**FIGURE 1:**
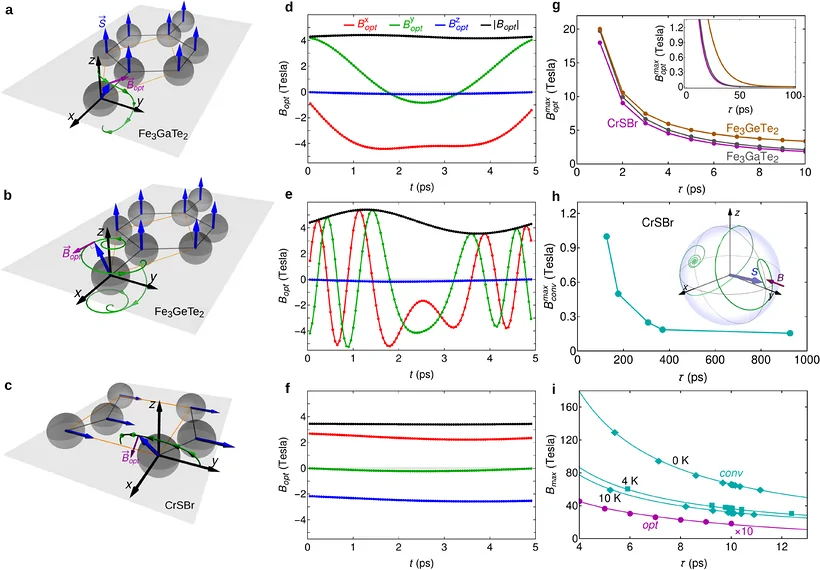
Magnetization switching in monolayer vdW magnets via optimal and conventional protocols. (a–c) Schematic representation of the magnetization switching in monolayer ${\mathrm{Fe}}_{3}{\mathrm{GaTe}}_{2}$, ${\mathrm{Fe}}_{3}{\mathrm{GeTe}}_{2}$, and CrSBr, respectively. The equilibrium orientations of the spins in each magnet ($\vec{s}$), corresponding to the local minima, are aligned along ±z for ${\mathrm{Fe}}_{3}{\mathrm{GaTe}}_{2}$ and ${\mathrm{Fe}}_{3}{\mathrm{GeTe}}_{2}$, and along ±y for CrSBr. Optimal control path between the energy minima is shown by green lines. The optimal external field ${\vec{B}}_{\mathrm{opt}}$ is shown via magenta arrows. The orange line displays the unit cell. (d–f) Optimal control field ($B_{\mathrm{opt}}$) as a function of time for τ=5 ps required to induce the transitions shown in (a)–(c). (g) Magnitude of maximum of switching field amplitude ($B_{\mathrm{opt}}^{\max}$) as a function of τ for all magnets with size of 50×50 ${\mathrm{nm}}^{2}$. The inset shows the fitted data points for τ>10 ps. (h) Maximum field amplitude for conventional switching ($B_{\mathrm{conv}}^{\max}$) in CrSBr with system size of 50×50 ${\mathrm{nm}}^{2}$. The applied field is fixed antiparallel to the initial spin orientation, as illustrated in the inset. (i) Maximum field required for magnetization switching as a function of τ, comparing optimal and conventional protocols at various temperatures. Values have been re‐scaled for clarity.

We also find that the optimal switching field $B_{\mathrm{opt}}$ exhibits its largest amplitudes during the initial half of the reversal (∼2.5 ps), where relaxation opposes the switching process. As the spins approach the equatorial point, relaxation reverses its role, naturally steering the system toward the final energy minimum (see Figure S4 and additional discussions). Throughout the process, the optimal field remains strictly perpendicular to the instantaneous magnetization (purple arrow in Figure 1a–c) and displays symmetric amplitudes that satisfy ${\vec{B}}_{\mathrm{opt}}\left(0\right)={\vec{B}}_{\mathrm{opt}}\left(\tau \right)$ [66, 67]. This trajectory resembles precessional switching but is reshaped by optimal control constraints. Extending the switching time further enhances the oscillatory character of the control paths around the anisotropy axis, together with the corresponding optimal pulses (Figures S1–S3). It is noteworthy that the optimal control field shown in Figure 1d–f is a chirped, circularly polarized microwave field combined with a bias component along the anisotropy axis. Such field configuration is closely connected to experimental techniques that are already well established. For instance, rotating microwave fields have long been used to control magnetization dynamics [68, 69, 70], and recent advances in microwave and photonic engineering allow precise control over both amplitude and frequency, including rapid chirping and complex temporal profiles [71, 72]. Moreover, the combination of such time‐dependent excitation with a static or slowly varying bias magnetic field is also routinely achieved in experiments [25, 73, 74], and even sophisticated shaping of magnetic field pulses has been demonstrated [4]. A possible experimental setup for the application of such optimized fields in vdW magnets is discussed in Section S3.

### 2.2 Conventional Switching and Thermal Effects

To benchmark the efficiency of the OCT protocol at different switching times τ, we compared the maximum field amplitudes required for optimal reversal ($B_{\mathrm{opt}}^{\max}$) with those of conventional switching ($B_{\mathrm{conv}}^{\max}$), as obtained from atomistic spin‐dynamic simulations (see Methods for details). In all three vdW magnets, $B_{\mathrm{opt}}^{\max}$ decreases systematically with increasing τ (Figure 1**g**), reflecting differences in anisotropy and intrinsic magnetic response. CrSBr consistently exhibits the lowest $B_{\mathrm{opt}}^{\max}$, remaining far below the other materials even at τ>10 ps (inset in Figure 1g), and was therefore selected as a model system for a detailed comparison. Figure 1h,i reveals that conventional switching requires significantly larger fields than OCT to achieve reversal on the same timescale. For example, at τ=126 ps, the static antiparallel field needed in the conventional scheme is 1 T, whereas OCT achieves switching with only 150 mT (Figure 1g–h). This stark contrast highlights fundamentally different pathways: conventional protocols rely on a fixed field and delayed precession before reversal, while OCT actively shapes the trajectory to minimize both amplitude and energy cost. Although both approaches predict a decrease in the peak field with increasing τ (Figure 1i), only OCT maintains low amplitudes across technologically relevant sizes, whereas conventional switching shows appreciable reductions only for much larger lateral dimensions. With industry roadmaps targeting progressively smaller magnetic bits [75], the superior scalability of OCT makes it particularly promising for next‐generation low‐power memory and logic technologies.

Thermal effects strongly influence the performance of conventional field‐driven switching. As the temperature increases, thermal activation lowers the coercive barrier and reduces the field strength needed for reversal [7] (Figure 1i). However, while higher temperatures such as 10 K continue to lower the average switching field, the increased thermal energy induces severe magnon scattering and dephasing. This disrupts the uniform precessional mode and introduces a massive statistical variance in the individual reversal trajectories, making the exact stabilization time highly unpredictable (see additional analysis in Section S4). In contrast, the OCT protocols, although designed at 0 K, already minimize the required field amplitudes and remain far below the conventional thresholds across all timescales examined. At ultrashort times (τ=4 ps), conventional switching demands fields exceeding 160 T at 0 K, whereas OCT achieves a successful reversal with only 4.6 T. Even at technologically relevant latencies (τ∼2 ns), OCT reduces the peak field to 9.6 mT compared to 329 mT for conventional protocols. This dramatic reduction illustrates the core advantage of OCT. By shaping the rotation of magnetic moments, it minimizes the external pulse needed and takes advantage of the intrinsic dynamics of the system to provide low‐power magnetization switching [67].

To place our results in context, we benchmark them against established NiFe‐based systems. Conventional spin valves switch within 140 ps with field pulses of 15.5 mT field [76], while other NiFe geometries require even longer times (200–4000 ps) at 4.5–10 mT [4, 68, 77]. Although the OCT protocol achieves reliable switching in CrSBr at 140 ps with a maximum field of only 135 mT, which might be larger than those mentioned in NiFe‐based devices, the primary goal is energy efficiency. Crucially, the aim of OCT is not merely to minimize peak‐field amplitudes but to reduce the total energy dissipated through Joule heating during reversal, yielding a fundamentally different optimization pathway. If OCT, however, was designed to reduce the field‐pulse amplitude instead of heat dissipation, it would consequently outperform conventional approaches to the detriment of energy savings (see Methods). Both OCT and conventional protocols produce coherent magnetization switching on the subpicosecond time scale (Figures S1–S3 and S6), but OCT does so with reasonably low fields, opening routes to more sustainable magnetic memory technologies.

### 2.3 Energy‐Efficient Switching

Having established that optimally shaped field pulses can reliably induce magnetization reversal, we next quantify the associated energetic cost. A central advantage of OCT is its inherent minimization of Joule heating [66, 67], which produces more energy‐efficient switching pathways than conventional schemes. In Figure 2a–d, we map the minimum switching energy $\Phi_{m}$ as a function of τ, system size, and temperature for conventional and OCT protocols. For the optimal case, $\Phi_{m}^{\mathrm{opt}}$ is extracted by inserting the computed OCP into Equation (8), while for the conventional case $\Phi_{m}^{\mathrm{conv}}$ is obtained by substituting the minimum static field required to trigger reversal at a given τ into Equation (2). Figure 2a shows $\Phi_{m}^{\mathrm{opt}}$ versus τ at 0 K for the three magnets with a fixed lateral size of 50×50 ${\mathrm{nm}}^{2}$, providing a direct comparison of energy costs under optimally adapted and traditional switching conditions. Across all materials, $\Phi_{m}^{\text{opt}}$ decreases monotonically with τ, consistent with the expected scaling $\Phi_{m}^{\text{opt}}∼\left|{\vec{B}}_{\text{opt}}\right|\tau$, where the amplitude of the optimal field $|{\vec{B}}_{\text{opt}}|$ decreases as τ increases (see Equation 5, Figure S7)[66, 67]. This behavior underscores a fundamental balance between speed and energy efficiency, offering a tunable control parameter for applications that demand ultrafast operation or minimal power consumption. Among the magnets studied, CrSBr consistently delivers the lowest energy cost, reinforcing its promise as a platform for highly energy‐efficient switching (Figure S8). The dependence of $\Phi_{m}^{\text{opt}}$ on the number of CrSBr layers is illustrated in Figure S9. We next examine conventional switching in CrSBr (Figure 2b). Here, $\Phi_{m}^{\mathrm{conv}}$ is plotted as a function of τ at 0 K for three different system sizes (30×50, 50×50, and 70×70 ${\mathrm{nm}}^{2}$). Although the qualitative trend mirrors that of optimal control, with energy costs decreasing as τ increases, the absolute values remain substantially higher across all sizes. This persistent gap highlights the inherent inefficiency of conventional field‐driven protocols in relation to optimally tailored pulses.

**FIGURE 2:**
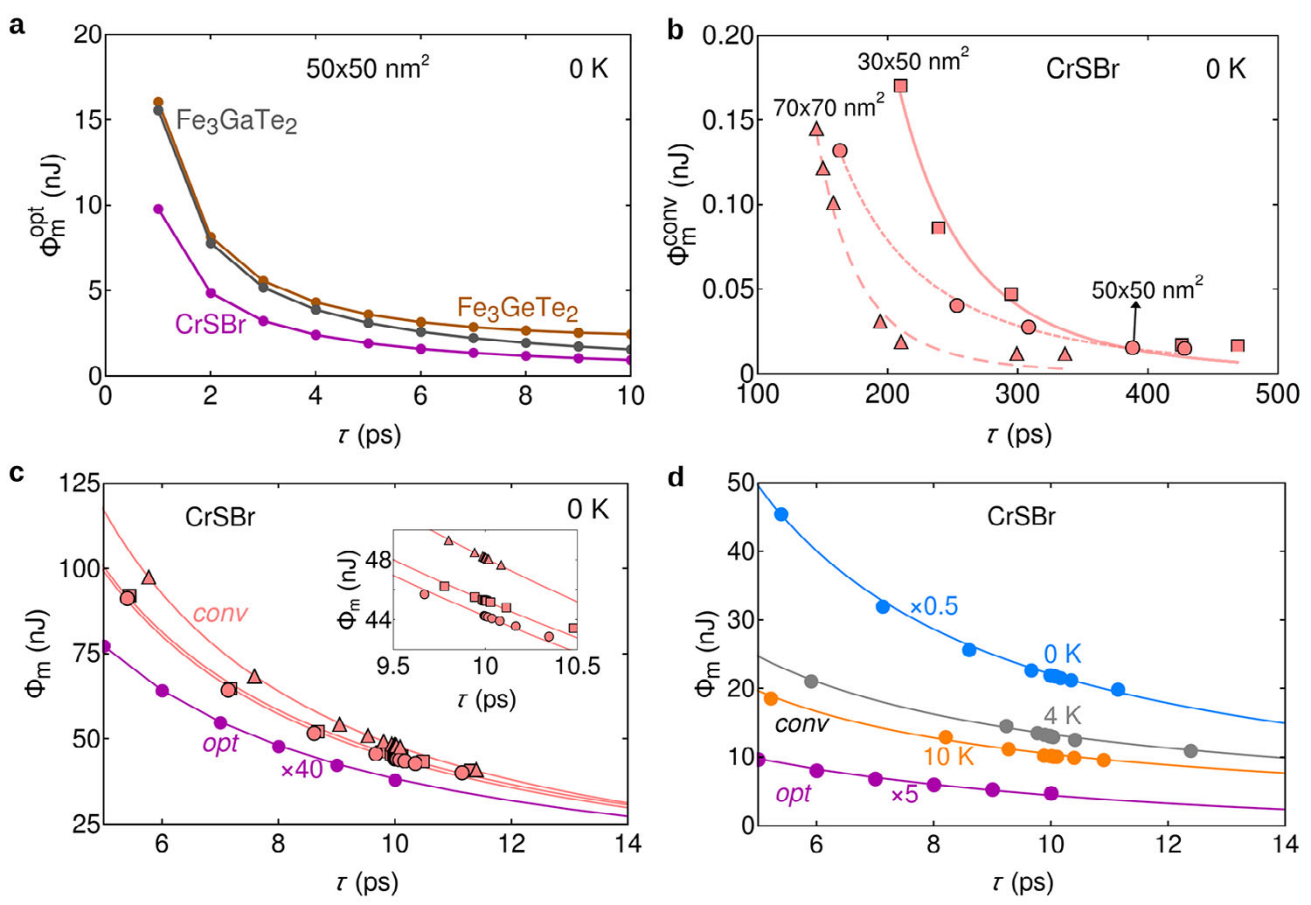
Energy cost of switching under optimal and conventional protocols. (a_) Minimum energy cost of magnetization switching using optimal control ($\Phi_{m}^{\text{opt}}$) at 0 K for monolayer ${\mathrm{Fe}}_{3}{\mathrm{GaTe}}_{2}$, ${\mathrm{Fe}}_{3}{\mathrm{GeTe}}_{2}$, and CrSBr, shown as a function of switching time τ, for a fixed system size of 50×50 ${\mathrm{nm}}^{2}$. (b) Minimum energy cost of conventional switching ($\Phi_{m}^{\text{conv}}$) in CrSBr at 0 K as a function of τ, shown for different system sizes. The data points are fitted with power laws of the form $\propto \tau^{b}$, with exponents b=−4.0, −2.5, and −4.5 corresponding to system sizes 30×50 ${\mathrm{nm}}^{2}$, 50×50 ${\mathrm{nm}}^{2}$, and 70×70 ${\mathrm{nm}}^{2}$, respectively. (c) Direct comparison between optimal (magenta, scaled up by a factor of 40) and conventional switching energy costs in CrSBr at 0 K. (d) Temperature dependence of conventional switching energies in CrSBr at 0 K (blue, scaled up by 0.5), 4 K, and 10 K, compared to optimal switching at 0 K (magenta, scaled up by 5).

To directly compare the energy efficiency of the optimal and conventional protocols, we plot the conventional switching data on shorter time scales and compare them with the optimal results in Figure 2c. The optimal data (scaled for clarity) correspond to CrSBr at 0 K with a size of 50×50 ${\mathrm{nm}}^{2}$, while the conventional curves reflect the size‐dependent results of Figure 2b. Throughout the full range of τ, OCT always yields significantly lower energy costs by more than an order of magnitude in the fast‐switching regime. This efficiency advantage persists even when the optimal field is constrained to zero temperature. We further assess the impact of finite temperatures on conventional switching in CrSBr (Figure 2d), comparing $\Phi_{m}^{\text{conv}}$ at 0, 4, and 10 K for a 50×50 ${\mathrm{nm}}^{2}$ system alongside the optimal switching result at 0 K (scaled for clarity). While thermal fluctuations lower the energy barriers for conventional field‐induced reversal, reducing $\Phi_{m}^{\mathrm{conv}}$, the energy cost remains substantially higher than that of the deterministic OCT pathway. Notably, even when conventional switching is aided by finite temperature, it fails to match the low energy cost achieved by the deterministic, non‐thermal optimal control pathway. Relative to cases where field‐driven magnetization reversal is considered efficient [76], such as spin valves switched by 140 ps magnetic pulses, the energy required (∼15 pJ) exceeds the 6.5 pJ projected for OCT‐driven CrSBr reversal on the same timescale. Similarly, switching a three‐terminal perpendicular magnetic tunnel junction (MTJ) through the spin‐orbit torque requires ∼0.4 pJ on 270 ps [78], whereas OCT achieves CrSBr reversal with 3 pJ in an equivalent window. These comparisons highlight that OCT not only minimizes energy dissipation already at zero temperature but also establishes a robust, scalable route for ultrafast, low‐power magnetization switching that surpasses conventional and current‐driven technologies alike.

### 2.4 Optimal Topological Path for Skyrmions

Up to this point, we have focused on optimal magnetization transitions between collinear states in vdW magnets. We now extend this framework to more complex, non‐collinear scenarios, exemplified by the collapse (or erasure process [79]) of an isolated skyrmion into a FM state in RT ${\mathrm{Fe}}_{3}{\mathrm{GaTe}}_{2}$ magnets. Applying OCT at zero temperature, we identify the OCP and analyze its characteristics relative to the conventional minimum‐energy path (MEP), which was likewise calculated at zero temperature and connects stable states along the lowest energy trajectory and whose saddle point sets the barrier for thermally activated transitions [80, 81] (see Methods for details). Comparing OCP and MEP highlights how control‐driven dynamics can access alternative pathways in configuration space, enabling ultrafast, low‐energy reversal.

Figure 3a shows the total energy difference relative to the FM state (E‐$E_{\mathrm{sky}}$) along the OCP for τ=10−20 ps (different curves) as a function of time t. Each trajectory exhibits three distinct regimes: (i) an initial slow energy buildup, (ii) a pronounced energy peak near the pulse midpoint, and (iii) a rapid relaxation toward the FM state. The consistent appearance of this peak indicates a common dynamical mechanism underlying skyrmion annihilation (Figure S10). Decomposition of E‐$E_{\mathrm{sky}}$ at a given τ (Figure 3b for τ=20 ps) into its several energy contributions (Dzyaloshinskii–Moriya interaction (DMI) ($E_{\text{DMI}}$), Heisenberg exchange ($E_{J}$) and anisotropy ($E_{K}$)) reveals that the energy peak coincides with a sharp increment in $E_{\text{DMI}}$ and $E_{J}$, which the latter combined with $E_{K}$ realign the spins toward the FM state and collapse the chiral spin textures. These changes are particularly sharp during the transition from configuration (iii) to (iv). Snapshots of the spin configuration using OCT (Figure 3e) illustrate this process, with Movie S4 displaying the entire process dynamically. The skyrmion initially stabilized near zero field [60] (Figure 3e(i)) undergoes radial collapse under the optimal pulse (Figure 3e(ii–iii)) where the spin texture shrinks preserving rotational symmetry. The subsequent steps (Figure 3e(iii–iv)) involved the emission of spin waves generated by energetic instabilities of the skyrmion core, driving the annihilation of the residual magnetic structure and coinciding with the energy peak (Figure 3b). Finally, the system relaxes to a uniform FM state through continued spin‐wave emission (Figure 3e(v–vi). This analysis demonstrates that OCT can orchestrate complex, non‐collinear spin textures along energy‐efficient, deterministic pathways, revealing dynamic routes inaccessible via conventional minimum‐energy considerations. The dependence of the annihilation cost on skyrmion radius is shown in Figure S11.

**FIGURE 3:**
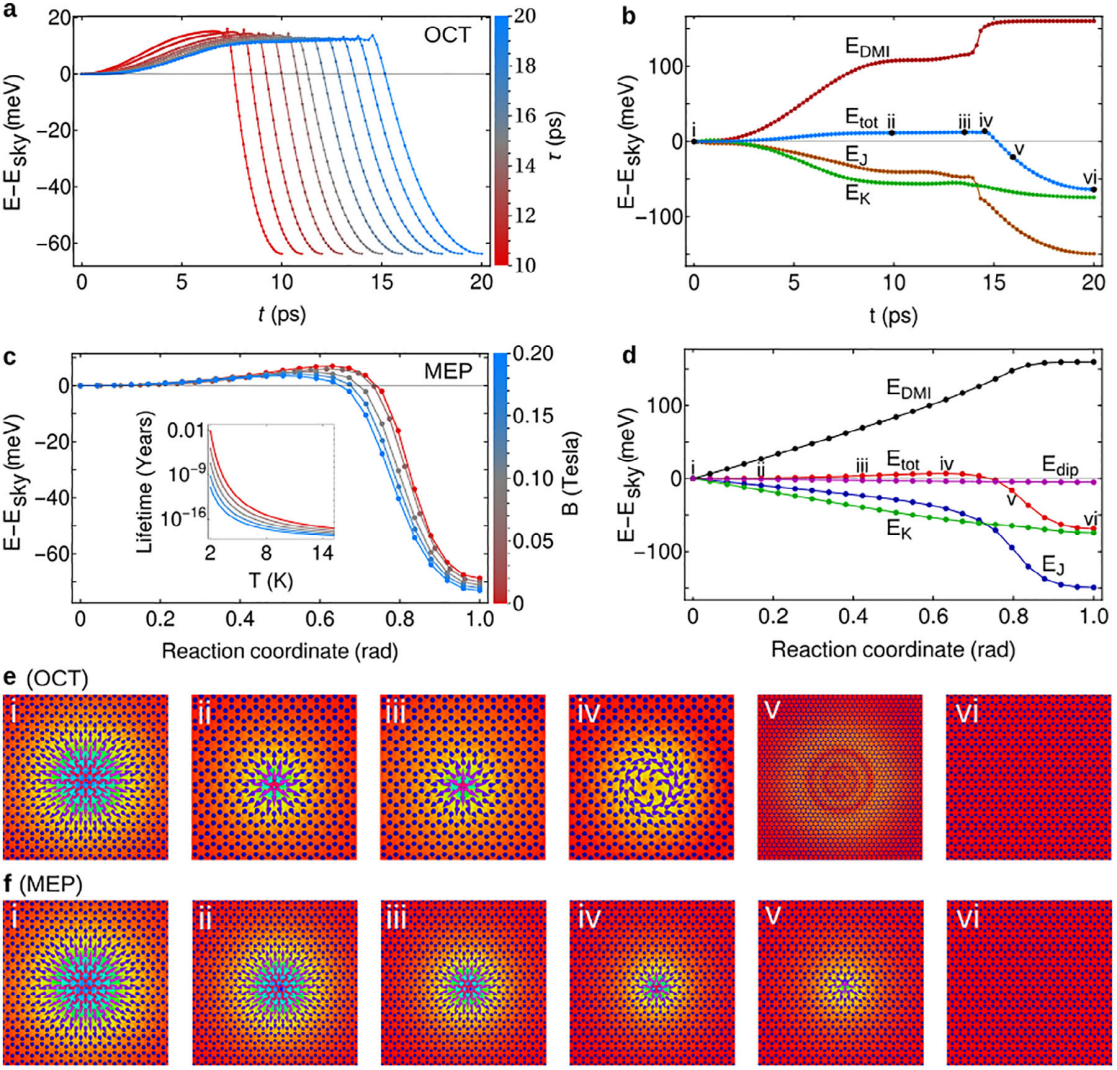
Transition dynamics of topological spin textures. (a) Variation of the total energy (E‐$E_{\mathrm{sky}}$) using OCT during skyrmion‐to‐FM transition in monolayer ${\mathrm{Fe}}_{3}{\mathrm{GaTe}}_{2}$ for different values of switching time τ (color bar). (b) Energy‐decomposition of E‐$E_{\mathrm{sky}}$ for different contributions (DMI, exchange, anisotropy) τ=20 ps. (c) Minimum energy path (MEP) for the skyrmion collapse or erasure for different external field values (color bar). The reaction coordinate is defined as the normalized displacement along the MEP. The inset shows the calculated lifetime (years) as a function of temperature (T). (d) The energy contribution of different interactions via E‐$E_{\mathrm{sky}}$ along the MEP at B=0 T. The OCT and MEP calculations were performed at T=0 K. (e) Selected reaction coordinates for the skyrmion evolution in OCT. Labels refer to their positions in (b). (f) Similar as in (e), but for labels referring to (d).

To contrast the field‐driven OCT dynamics with the intrinsic thermally activated behavior, we computed the MEP using the geodesic nudged elastic band (GNEB) method (see Methods for details). Figure 3c shows the MEP energy profile as a function of the reaction coordinate for different external field values, referenced to the skyrmion state. While the MEP lacks explicit time dependence, its energy landscape closely resembles that observed along the OCT trajectory. As the external field increases, the skyrmion radius decreases, lowering the energy barrier [82, 83], consistent with transition‐state predictions of reduced lifetimes at higher temperatures (inset in Figure 3c). The decomposition of the MEP energy via E‐$E_{\mathrm{sky}}$ (Figure 3d) reveals a monotonic increase in DMI as the system unfolds toward the FM state, while the contributions to $E_{J}$ and $E_{K}$ follow trends similar to those observed in OCT‐driven dynamics (Figure 3b). Snapshots along the MEP (Figure 3f(i–vi) and Movie S5) illustrate a symmetric collapse pathway without spin wave excitations, in contrast to the richer dynamical features of the OCT trajectory. Interestingly, the peak energy along the MEP is lower than the maximum along the OCP (Figure 3a), indicating that minimizing the total switching energy through optimal control does not necessarily coincide with lowering the energy barrier between the initial and final states [66, 67]. The evolution of the topological charge during skyrmion annihilation along both MEP and OCP is provided in Figure S12, further highlighting the distinct dynamical pathways accessible through control‐driven versus thermally activated processes (see Figure S13).

### 2.5 Benchmark Against Different Technologies

To establish a quantitative benchmark for the minimum energy cost of magnetization transition $\Phi_{m}^{\text{opt}}$ with state‐of‐the‐art memory technologies, we applied OCT to two cases: (i) magnetization reversal in a 5×5 ${\mathrm{nm}}^{2}$ CrSBr‐like vdW magnet for different damping values α ranges from 0.001 up to 0.05, and (ii) isolated skyrmion annihilation (or erasure) in a 50×50 ${\mathrm{nm}}^{2}$ ${\mathrm{Fe}}_{3}{\mathrm{GaTe}}_{2}$ vdW magnet hosting a skyrmion of radius ∼2.5 nm (Figure 3). Note that in OCT, α plays a substantial role in the switching process, whereas other parameters assume a minor contribution. Thus, variations of other quantities (exchange, anisotropy, DMI, etc.) for CrSBr have not been included here. The resulting $\Phi_{m}^{\text{opt}}-\tau$ maps (Figure 4a,b) expose the fundamental energy–time interplay that constrains optimal magnetic switching in the fJ scale. We first consider the multi‐nanosecond switching (Figure 4a), where a markedly different scaling emerges. For coherent reversal in the CrSBr‐like magnet, $\Phi_{m}^{\text{opt}}$ decreases steadily from ≈9 fJ at 6 ns, to ≈2.4 fJ at 20 ns for α=0.001, and a clear separation between different damping constants emerges beyond τ≥4 ns as internal dynamics begin to control the process. In contrast, skyrmion annihilation becomes increasingly inefficient at these longer times (by a factor of 10), exceeding the optimal cost of coherent reversal in all α’s. At τ=10 ns, the OCT skyrmion protocol yields $\Phi_{m}^{\text{opt}}\approx$60 fJ, comparable to the experimentally reported ≈100 fJ for skyrmion annihilation using chiral magnetic tunnel junction [84], but orders of magnitude below the $3\times 10^{4}$ and $5\times 10^{5}$ fJ energies typically needed for skyrmion nucleation using current pulses [85, 86], and to write a bit in commercial NOR flash memories ($10^{6}$ fJ)[87]. These results show that OCT can reveal not only optimal trajectories connecting different metastable or ground states, but also the crossover between texture‐dependent dynamical regimes.

**FIGURE 4:**
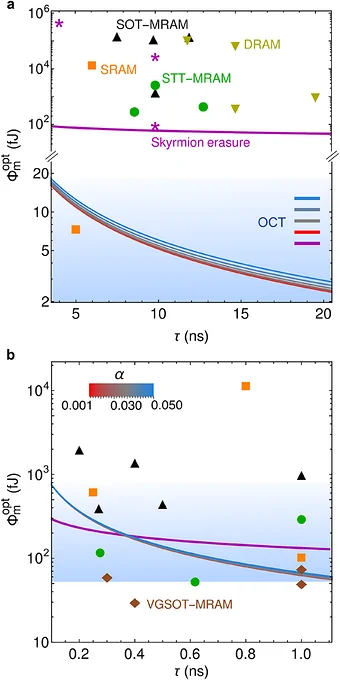
Optimal switching energy compared with state‐of‐the‐art memory technologies. Minimum energy cost $\Phi_{m}^{\text{opt}}$ as a function of switching time τ (solid lines) for a 5×5 ${\mathrm{nm}}^{2}$ monolayer CrSBr‐like magnet and skyrmion annihilation in monolayer ${\mathrm{Fe}}_{3}{\mathrm{GaTe}}_{2}$ (magenta line). Results are shown in the multi‐nanosecond range (a) and sub‐nanosecond range (b) for different values of the Gilbert damping parameter α, color‐coded as indicated in the legend bar. The highlighted blue areas in (a) and (b) correspond to the energy range where OCT is mostly efficient across switching times. Symbols show comparison with available experimental data across different technologies: SRAM (orange) [87, 96, 97, 98, 99], DRAM (yellow) [87, 147, 148], STT‐MRAM (green) [91, 92, 93, 94, 95], SOT‐MRAM (black) [78, 88, 89, 90, 91], and VGSOT‐MRAM (brown) [88, 100, 101, 102]. Skyrmion erasure label refers to magenta stars and curve [84, 85, 86]. Section 14 includes the summary of all data extracted across different technologies.

In the sub‐nanosecond regime (τ<1 ns), the cost of reversal is dominated by the need for strong driving fields and shows only weak sensitivity to α (e.g., the curves are mostly superposed to each other), with $\Phi_{m}^{\text{opt}}$ of a few hundreds of femtojoules (e.g., 356 fJ at τ=0.2 ns) falling below 100 fJ at τ≈1 ns (Figure 4b). In contrast, skyrmion erasure initially (τ<0.4 ns) requires less energy than uniform reversal, reflecting the efficiency of destabilizing a localized texture, but this advantage disappears for longer switching times as topological constraints become dominant and provide additional stability to spins.

Benchmarking against existing technologies underscores the performance gap (Tables S1–S6). Across several different compounds and systems, the reported switching energies for SOT‐MRAM (Figure 4a,b) span 450−2000 fJ in the sub‐nanosecond regime and can exceed 10 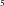 fJ at longer times [78, 88, 89, 90, 91], while STT‐MRAM often requires ∼54− 300 fJ at 0.3 − 1 ns and several pJ's for slower switching [91, 92, 93, 94, 95]. Static random access memory (SRAM) lies in the 100−600 fJ range at 0.25–1 ns but also rises steeply at longer times [87, 96, 97, 98, 99]. Dynamic random‐access memory (DRAM) is mostly observed within 3.9 × 10^2^ − 1.1 × 10^5^ fJ in 12−20 ns, which is 3−6 orders of magnitude higher in energy than any of the OCT results (Figure 4a). In contrast, VGSOT reaches 3075 fJ at 0.3−1 ns [88, 100, 101, 102], a level matched by OCT protocols at τ∼1 ns. The same OCT framework also refers to skyrmion‐based neuromorphic concepts, where thermal nucleation demands ∼20 pJ per skyrmion [103] and voltage control of magnetic anisotropy (VCMA) can push below 100 fJ [84], approaching the 1−100 aJ range estimated for biological synapses [104] and far below the ∼12.7 pJ per event in CMOS neuromorphic chips [104]. We observed that much lower energy regimes can be achieved by OCT at slightly longer times, such as 1.91 fJ at τ=25 ns for systems with low damping (α=0.001), which is one order of magnitude lower than the synaptic processes involved in vesicle release (∼25 fJ)[105]. Thus, OCT protocols not only define the theoretical limits of energy‐efficient memory switching but also exceed the energy‐efficiency scale of neuromorphic and biological systems.

### 2.6 On the Experimental Realization of OCT

In practice, this switching process would require that at least two components of the field be optimized, which are orthogonal to the easy‐axis of the magnet. Thus, one field component drives the precession (e.g., $B_{\mathrm{opt}}^{x}$ in CrSBr (Figure 1f)), whereas another fine‐tunes the trajectory ($B_{\mathrm{opt}}^{z}$). In this case, $B_{\mathrm{opt}}^{x}$ is often the cleanest control knob to induce precessional motion while staying close to the plane of the device. In many strip line geometries, this is also the most natural pulsed field component to generate reproducibly [4, 69, 71] and provide the torque required to initiate precessional reversal (Figure S4). The co‐optimization of $B_{\mathrm{opt}}^{z}$ will tilt the orbit, accelerate escape from the initial minimum, and help suppress back‐switching or ringing. Effectively, even if $B_{\mathrm{opt}}^{z}$ is not intentionally generated, some fringing or nonuniform pulse field may create it anyway. Sometimes that parasitic component actually helps and should be engineered rather than eliminated. The optimum component of the field along the easy‐axis of the magnet ($B_{\mathrm{opt}}^{y}$ for CrSBr) can reshape the barrier between states or bias the final state, but by itself it is not the efficient component for launching precession from an initially collinear state. Hence, for pure pulse optimization, $B_{\mathrm{opt}}^{y}$ is usually secondary.

A possible experimental setup for the application of such optimized fields would follow a combination of established platforms [4, 10, 25, 73, 74] with the magnetic layer deposited on top of an on‐chip transmission line [4, 25]. A nearby microcoil, strip line, or coplanar waveguide might work, with the field source as close to the active magnetic region as possible so that a modest current generates a strong local field with high spatial efficiency. Ultrafast excitation is generated using either a broadband arbitrary waveform generator combined with a high‐bandwidth pulse amplifier [72] or a photoconductive switch (e.g., Auston‐type [106]) triggered by a femtosecond laser for picosecond pulse generation. A bias‐tee configuration allows simultaneous application of static and dynamic fields, while careful impedance matching and microwave design minimize reflections and energy loss. The magnetic response is probed using time‐resolved pump–probe techniques, such as the magneto‐optical Kerr effect (TR‐MOKE) [38, 107, 108] with sub‐picosecond temporal resolution, or by electrical readout through anisotropic or tunnel magnetoresistance in device‐integrated geometries. To quantify energy dissipation, the voltage and current delivered to the device are measured in situ using high‐bandwidth oscilloscopes and calibrated directional couplers, enabling direct evaluation of the injected pulse energy. The field geometry is engineered in a local anisotropy frame, where a dominant transverse in‐plane component is generated by the current pulse in the strip line, while smaller out‐of‐plane and longitudinal components are tuned via device geometry and external bias fields. The experiment is operated in a closed‐loop configuration in which the pulse parameters (amplitude, duration, waveform shape, and relative phase) are iteratively adjusted using data‐driven optimization algorithms (Bayesian or reinforcement learning [109, 110, 111]) to minimize the total energy required for deterministic switching. This integrated hardware–software architecture provides a versatile and scalable route to experimentally identify optimal low‐energy switching protocols under realistic device and thermal conditions.

In terms of device applicability, it is important to note that within the OCT framework, the shape of the derived magnetic fields is dictated by the spin dynamics governed by the Landau–Lifshitz–Gilbert (LLG) equation. In fact, the derived optimal fields reflect the synchronization of the external torque with the precessional motion and damping of the magnetization in the material [4, 25, 112]. It is noteworthy that the magnetic fields remain smooth, continuous, and bounded, with characteristic frequencies set by the intrinsic dynamical scales of the system. In this context, the OCT approach does not imply the need for device‐specific or continuously re‐optimized control protocols. For a given magnet and geometry, optimal solutions are primarily determined by intrinsic material parameters such as magnetic anisotropy, damping, and saturation magnetization, leading to reproducible and transferable pulse profiles. It is worth mentioning that the formula used to derive the optimal fields depends on the dynamical trajectory of the system and the effective field (see Equation 5). Furthermore, time‐dependent control is already inherent in magnetization switching schemes driven by tailored external magnetic fields, where shaped pulses are routinely used to enhance switching efficiency [4, 25, 112].

In possible applications using on‐chip memory technologies, the control space can be restricted to low‐dimensional pulse families that are already standard on industrial platforms, such as single or double pulses, trapezoidal waveforms, or short resonant bursts with tunable amplitude and timing [4, 18, 76, 113, 114, 115]. OCT can then be applied within this constrained space to determine optimal parameter sets (pulse amplitude, duration, delay, and phase) that minimize switching energy while preserving deterministic operation. Importantly, this results in a small library of reusable pulse templates, rather than device‐specific waveforms, which can be uniformly applied across large arrays of nominally identical memory cells. From a more hardware perspective, such pulse schemes are compatible with existing and emerging RF‐CMOS and spintronic driver circuits [116, 117], which routinely support programmable pulse shaping, pre‐emphasis, and multi‐level signal generation at GHz bandwidths. Similar concepts have already been deployed in high‐speed interconnects and magnetic recording technologies [118, 119, 120, 121], where modest waveform shaping significantly improves energy efficiency and signal fidelity without compromising scalability. OCT plays a role analogous to offline calibration or design optimization, rather than an operational overhead.

Moreover, device variability and thermal fluctuations, which are critical in high‐density memory arrays [120, 121], can be naturally incorporated into the optimization loop, enabling the identification of robust pulse protocols that tolerate fabrication spread and noise. Once determined, these protocols can be implemented either as fixed waveforms or as a small set of selectable modes (e.g., fast/low‐energy write), without requiring per‐bit adaptive control. We therefore state that the proposed approach is compatible with scalable memory architectures, as it leverages OCT to discover simple, bandwidth‐limited, and hardware‐efficient pulse schemes that can be directly integrated into existing write‐driver circuitry, rather than requiring complex, continuously varying field control.

## 3 Conclusion

We have developed and validated an optimal control framework for designing ultrafast, low‐dissipation magnetization transitions in vdW magnets. By replacing heuristic scans over pulse amplitudes and frequencies with full optimal control pathways, time‐resolved trajectories through configuration space, this approach directly connects initial and target states while minimizing energy loss. Applied to ${\mathrm{Fe}}_{3}{\mathrm{GaTe}}_{2}$, ${\mathrm{Fe}}_{3}{\mathrm{GeTe}}_{2}$, and CrSBr, it produces sub‐picosecond switching at a critically reduced energy cost compared to conventional protocols and automatically tailors pulse shapes to the intrinsic properties of each material. Extending the methodology to topologically non‐trivial textures such as skyrmions reveals not only the minimum‐energy routes for annihilation but also the spin‐wave excitations that accompany their collapse, providing a quantitative handle on complex, non‐equilibrium spin dynamics.

Beyond demonstrating a clear reduction in the energy cost of magnetic switching, this formalism opens a pathway to material‐specific, programmable spintronics operations. By using the optimal control pulses as inverse design templates, one could embed energy‐efficient switching directly into memory or logic elements based on 2D magnets, enabling on‐chip manipulation of bits at picosecond speeds. The framework also offers a predictive tool for benchmarking the ultimate limits of switching energies in emerging spintronics architectures, including neuromorphic platforms where biological‐level energy efficiency is sought. A key advance now within reach is the incorporation of finite‐temperature OCT, in which the cost functional explicitly accounts for thermal fluctuations and noise. Such an extension would exploit temperature not merely as a perturbation but as a resource, dynamically steering the system along stochastic but lower‐barrier trajectories and thereby reducing the external torque required for reversal. By jointly optimizing the field (or current) waveform and the statistical ensemble of trajectories, the formalism could predict pulse shapes that are robust to thermal activation, yet exploit it to minimize dissipation, particularly in technologically relevant regimes where devices operate well above cryogenic temperatures.

Even though the present study focuses on externally applied magnetic fields, the same mathematical machinery can be straightforwardly extended to current‐driven torques (SOT, STT, or voltage‐controlled magnetic anisotropy) where the control variables become electric currents or gate voltages rather than fields. Combined with finite‐temperature OCT, this would enable pulse shaping of current waveforms that simultaneously harness thermal assistance and minimize Joule heating and stray fields while exploiting the local symmetry of device stacks. Such an extension could accelerate the translation of optimal control from theory to practice, offering a unifying route to deterministic, low‐power manipulation of nanoscale magnetic states across both field‐driven and current‐driven spintronics technologies.

## 4 Methods

### 4.1 Energy Cost of the Magnetization Transition

We define the cost of the magnetization transition as the amount of energy used to generate the control pulse that produces the desired change in the magnetic structure of the system [66, 67, 122]

(1) $$\Phi =\int_{0}^{\tau }\int_{V}{\left|\vec{B}\left(\vec{r},t\right)\right|}^{2}Rdtd\vec{r},$$

where τ is the switching time, V is the volume of the magnetic sample, $\vec{B}\left(\vec{r},t\right)$ is the external magnetic field generated at site $\vec{r}$ and time t, and R is the resistance of the device generating B. Here we set R=1Ω and assume that it remains approximately constant as the magnetization direction evolves. The purpose of the OCT is to identify the optimal pulse ${\vec{B}}_{\mathrm{opt}}\left(\vec{r},t\right)$ that brings the system to the target state such that Φ is minimized. Although it is theoretically possible to develop an OCT formalism with both energy cost and field amplitude being minimized simultaneously, it would require substantial efforts beyond the work included here. In a discrete model, spatial integration over a continuous system in Equation (1) is replaced by a summation over the lattice sites where the magnetic moments are localized, yielding the following expression for the cost functional:

(2) $$\Phi =c\int_{0}^{\tau }\sum_{i=1}^{N}{\left|{\vec{B}}_{i}\left(t\right)\right|}^{2}dt,$$

where the factor c is proportional to the volume of the unit cell and N is the total number of magnetic moments. In the following, we consider c=1.

Whenever thermal fluctuations are negligible, the system dynamics can be described by the LLG equation [123]:

(3) $$\left(1+\alpha^{2}\right)\frac{d{\vec{s}}_{i}}{dt}=-\gamma {\vec{s}}_{i}\times \left({\vec{b}}_{i}+{\vec{B}}_{i}\right)-\alpha \gamma {\vec{s}}_{i}\times \left[{\vec{s}}_{i}\times \left({\vec{b}}_{i}+{\vec{B}}_{i}\right)\right],$$

where ${\vec{s}}_{i}$ is the ith normalized magnetic moment vector, γ is the gyromagnetic ratio, α is the damping factor, and ${\vec{b}}_{i}$ is the internal magnetic field defined by the magnetic configuration through the following equation:

(4) $${\vec{b}}_{i}=-\frac{1}{\mu_{i}}\frac{\partial E}{\partial {\vec{s}}_{i}}$$

with $\mu_{i}$ being the ith magnetic moment length and E the internal energy of the system.

Both ${\vec{B}}_{i}\left(t\right)$ and ${\vec{s}}_{i}\left(t\right)$ can be treated as independent variables, and Φ minimized subject to the constraint defined by Equation (3) [124, 125]. Alternatively, the optimal pulse ${\vec{B}}_{i}^{m}\left(t\right)$ can be calculated via the unconstrained minimization of Φ. For this, Equation (3) is used to express the external magnetic field through the dynamical trajectory and the internal magnetic field [66]:

(5) $$\begin{array}{c} {\vec{B}}_{i}\left({\vec{s}}_{i},{\dot{\vec{s}}}_{i}\right)=\frac{\alpha }{\gamma }{\dot{\vec{s}}}_{i}+\frac{1}{\gamma }\left[{\vec{s}}_{i}\times {\dot{\vec{s}}}_{i}\right]-{\vec{b}}_{i}^{⊥}, \end{array}$$

and the result substituted into Equation (2). Subsequently, the energy cost Φ becomes a functional of the switching trajectory M:

(6) $$\Phi =\Phi \left[M\right]=\int_{0}^{\tau }\sum_{i=1}^{N}A\left({\vec{s}}_{i},{\dot{\vec{s}}}_{i}\right)dt,$$

where $M:=({\vec{s}}_{1},\text{…},{\vec{s}}_{N})$ is a 3N dimensional vector and $A({\vec{s}}_{i},{\dot{\vec{s}}}_{i})$ is given by

(7) $$A\left({\vec{s}}_{i},{\dot{\vec{s}}}_{i}\right)=\frac{\alpha^{2}+1}{\gamma^{2}}|{\dot{\vec{s}}}_{i}{|}^{2}-\frac{2\alpha }{\gamma }{\dot{\vec{s}}}_{i}\cdot {\vec{b}}_{i}^{⊥}-\frac{2}{\gamma }\left({\vec{s}}_{i}\times {\dot{\vec{s}}}_{i}\right)\cdot {\vec{b}}_{i}^{⊥}+{\left|{\vec{b}}_{i}^{⊥}\right|}^{2}.$$

### 4.2 Numerical Optimization of the Energy Cost Functional

We find OCPs numerically via direct minimization of the cost functional. For this, we discretize Φ using the midpoint rule

(8) $$\Phi \left[M\left(t\right)\right]\approx \Phi \left[S\left(t\right)\right]=\sum_{p=0}^{Q}\sum_{i=1}^{N}{\left|{\vec{B}}_{p+\frac{1}{2},i}\right|}^{2}\left(t_{p+1}-t_{p}\right),$$

where $\left\{t_{p}\right\}$ is a partition of the time interval [0,τ] such that $0=t_{0}<t_{1}<\text{…}<t_{Q+1}=\tau$. Here, the partition has a regular spacing, that is, $t_{p+1}-t_{p}=\Delta t=\tau /\left(Q+1\right)$, p=0,…,Q. A switching trajectory M(t) is represented by a polygeodesic line connecting Q+2 points, referred to as ‘images’: $M\left(t\right)\to \left\{M_{0},M_{1},\text{…},M_{Q+1}\right\}$, with $M_{p}:=\left\{{\vec{s}}_{p,1},\text{…},{\vec{s}}_{p,N}\right\}$ and ${\vec{s}}_{p,i}={\vec{s}}_{i}\left(t_{p}\right)$. The first image $M_{0}$ and the last image $M_{Q+1}$ correspond to the initial and the final orientation of the magnetic moments, respectively; they are fixed, but Q intermediate images can be moved (Figures S14). The external field ${\vec{B}}_{p+\frac{1}{2},i}\equiv \vec{B}\left({\vec{s}}_{p+\frac{1}{2},i},{\dot{\vec{s}}}_{p+\frac{1}{2},i}\right)$ is defined by the position and velocity of the magnetic moments at the midpoint of the discretization intervals via Equation (5). On the other hand, both ${\vec{s}}_{p+\frac{1}{2},i}$ and ${\dot{\vec{s}}}_{p+\frac{1}{2},i}$ can be expressed via the position of the images:

(9) $$\begin{array}{cc} {\vec{s}}_{p+\frac{1}{2},i} & =\frac{{\vec{s}}_{p+1,i}+{\vec{s}}_{p,i}}{|{\vec{s}}_{p+1,i}+{\vec{s}}_{p,i}|}, \end{array}$$

(10) $$\begin{array}{cc} {\dot{\vec{s}}}_{p+\frac{1}{2},i} & =\frac{\delta_{p,i}}{\Delta t}\frac{{\vec{s}}_{p+1,i}-{\vec{s}}_{p,i}}{|{\vec{s}}_{p+1,i}-{\vec{s}}_{p,i}|}, \end{array}$$

where $\delta_{p,i}$ is the angle between ${\vec{s}}_{p,i}$ and ${\vec{s}}_{p+1,i}$ (Figure S14). Upon substituting Equations (9), (10), and (5) into Equation (8), the functional Φ becomes a function of a 3QN−dimensional vector S defining the position of the movable images, $S=\left(M_{1},\text{…},M_{Q}\right)$.

Possible OCPs of magnetization switching can be identified by locating minima of Φ(S). This is done using the velocity projection optimization (VPO) method [126] and/or the limited‐memory Broyden‐Fletcher‐Goldfarb‐Shanno (LBFGS) algorithm [127, 128] equipped with the force acting on the movable images:

(11) $$F=-\nabla^{⊥}\Phi \left[S\right],$$

where $\nabla^{⊥}$ denotes the gradient projected on the tangent space of the configuration space, which is a curved manifold due to the constraint $|{\vec{s}}_{p,i}|=1$, p=1,…,Q and i=1,…,N. Explicitly:

(12) $${\vec{\nabla }}^{⊥}=\left({\vec{\nabla }}_{1,1}^{⊥},{\vec{\nabla }}_{1,2}^{⊥},\text{…},{\vec{\nabla }}_{1,N}^{⊥},{\vec{\nabla }}_{2,1}^{⊥},\text{…},{\vec{\nabla }}_{Q,N}^{⊥}\right),$$

where ${\vec{\nabla }}_{p,i}^{⊥}\equiv \partial /\partial {\vec{s}}_{p,i}-{\vec{s}}_{p,i}\left({\vec{s}}_{p,i}\cdot \partial /\partial {\vec{s}}_{p,i}\right)$.

For a given number of images involved in the local minimization of Φ(S), the calculation is considered converged when the magnitude of the force, |F|, has dropped below the set tolerance. In this work, up to Q=100 movable images were used depending on the system at hand, as well as the parameters of the system and the switching time, with the lowest force tolerance corresponding to the decrease of the force by eight orders of magnitude with respect to the initial force calculated from the initial path.

### 4.3 Atomistic Spin Model

We employed atomistic simulation methods using our in‐house codes and the VAMPIRE software [42, 47, 56, 57, 129, 130, 131, 132] to investigate the magnetic properties of 2D magnetic materials. The energy of the magnetic system is determined based on the spin Hamiltonian:

(13) $$\begin{array}{ccc} H & = & -\sum_{i,j}J_{ij}{\vec{s}}_{i}\cdot {\vec{s}}_{j}-\sum_{i,j}{\vec{D}}_{ij}\cdot \left({\vec{s}}_{i}\times {\vec{s}}_{j}\right)-\sum_{l}K_{l}\sum_{i}{\left({\vec{s}}_{i}\cdot {\hat{e}}_{l}\right)}^{2}\\ & & -\mu_{s}\sum_{i}{\vec{B}}_{i}\cdot {\vec{s}}_{i}+H_{d}, \end{array}$$

where summation in the first two terms runs over distinct pairs of magnetic atoms, ${\vec{s}}_{i}$ is a unit vector pointing along the ith magnetic moment whose magnitude is $\mu_{s}$ for each lattice site, the strength of the exchange interaction between pairs of normalized magnetic moments ${\vec{s}}_{i}$ and ${\vec{s}}_{j}$ at lattice sites i and j is characterized by the exchange constants $J_{ij}$. Additionally, DMI [133, 134] is described by the vectors ${\vec{D}}_{ij}=D_{ij}\left(\hat{z}\times {\vec{r}}_{ij}\right)$ with $\hat{z}$ and ${\vec{r}}_{ij}$ being the unit vector along the monolayer normal and the vector joining the i‐ and j‐th magnetic moments, respectively. The third term in Equation (13) signify the on‐site anisotropy, with $K_{l}$ being the anisotropy constants and ${\hat{e}}_{l}$ is the direction of the anisotropy axes. The fourth term is the Zeeman energy, where the optimal external magnetic field $\vec{B}\mathrm{opt}$ is calculated inversely using the OCT approach, while in the ASD simulations a static field $\vec{B}$ is applied antiparallel to the initial magnetization. The system parameters can be found in Section S11. The last term in Equation (13) corresponds to the long‐range dipole‐dipole interaction, defined by the equation:

(14) $$\begin{array}{c} H_{d}=\frac{\mu_{0}\mu_{s}^{2}}{4\pi }\sum_{i\neq j}\left[\frac{{\vec{s}}_{i}\cdot {\vec{s}}_{j}}{r_{i,j}^{3}}-\frac{3\left({\vec{s}}_{i}\cdot {\vec{r}}_{i,j}\right)\left({\vec{s}}_{j}\cdot {\vec{r}}_{i,j}\right)}{r_{i,j}^{5}}\right], \end{array}$$

with $\mu_{0}$ being the vacuum magnetic permeability. Calculation of $H_{d}$ is performed using the Fast Fourier Transform (FFT) [135, 136, 137] and macrocell [129, 138] approaches as implemented in our in‐house OCT code and VAMPIRE software, respectively.

### 4.4 Geodesic Nudged Elastic Band Method

We employ the VPO and/or orthogonal spin optimization combined with LBFGS [128] algorithms to obtain isolated soliton states corresponding to local energy minima of the Hamiltonian in Equation 13. The minimum energy path for their collapse is then determined using the geodesic nudged elastic band (GNEB) method [126]. The initial guess for the GNEB calculations is constructed as a geodesic path connecting the soliton configuration to the ferromagnetic state (Section S13). Both optimization and GNEB methods are implemented within the in‐house code.

### 4.5 Finite‐Temperature Atomistic Spin Dynamics

Interaction of the magnetic systems with the heat bath is described by including a stochastic term in the zero‐temperature LLG equation (Equation 3), resulting in stochastic LLG for the magnetic moment motion [123, 139, 140, 141]

(15) $$\frac{d{\vec{s}}_{i}}{dt}=-\frac{\gamma }{\left(1+\alpha^{2}\right)}\left[{\vec{s}}_{i}\times {\vec{H}}_{i}^{\mathrm{eff}}+\alpha {\vec{s}}_{i}\times \left({\vec{s}}_{i}\times {\vec{H}}_{i}^{\mathrm{eff}}\right)\right],$$

where ${\vec{H}}_{i}^{\mathrm{eff}}$ denotes the effective magnetic field acting on the i‐th magnetic moment as described by

(16) $${\vec{H}}_{i}^{\mathrm{eff}}=-\frac{1}{\mu_{i}}\frac{\partial H}{\partial {\vec{s}}_{i}}+{\vec{H}}_{i}^{\mathrm{th}},$$

with the first (second) term being the effective (fluctuating) field acting at the i‐th magnetic moment. Note that ${\vec{H}}_{i}^{\mathrm{th}}$ models the thermal fluctuations of the system in the Langevin dynamics framework [81]. Moreover, ${\vec{H}}_{i}^{\mathrm{th}}$ can be described using the Gaussian distribution thanks to the central limit theorem. Here, we assume that ${\vec{H}}_{i}^{\mathrm{th}}$ has the following properties: (i) ${\vec{H}}_{i}^{\mathrm{th}}$ are rotational invariant and do not favor any direction, i.e., the mean value of the distribution must vanish; (ii) the Cartesian components of ${\vec{H}}_{i}^{\mathrm{th}}$ are δ correlated in space and time [81]. The time evolution of the system within the simulation framework of atomistic spin dynamics is determined by numerical integration of the stochastic LLG equation using the implicit midpoint method, as implemented in the VAMPIRE software [129]. At each time step of the simulations, the instantaneous stochastic field acting on the i‐th ion position is given by

(17) $${\vec{H}}_{i}^{\mathrm{th}}=\vec{\Gamma }\left(t\right)\sqrt{\frac{2\alpha k_{B}T}{\gamma \mu_{i}\Delta t}},$$

where $\vec{\Gamma }\left(t\right)$ is a stochastic vector whose components follow a Gaussian distribution with zero mean and unit variance. The width of this distribution is scaled by the strength of the thermal effects. In this context, $k_{B}$ denotes the Boltzmann constant, T is the system temperature, and Δt is the integration time step [142, 143]. At each time step, three independent Gaussian random numbers are required per spin. In VAMPIRE, these are generated using the Mersenne Twister algorithm [144], followed by the Ziggurat method [145] to transform the uniform distribution into a Gaussian one. Each simulation consisted of three stages: (1) initial equilibration at zero applied magnetic field to establish a Boltzmann distribution [146]; (2) switching under the application of the static field, with thermal fluctuations included throughout this stage; and (3) final equilibration at zero applied magnetic field. It is important to emphasize that while thermal effects introduce stochastic fluctuations in spin moments ${\vec{s}}_{i}$ and consequently in their magnitudes $\mu_{i}$, they do not affect the system parameters defined in Equation (13). To ensure statistical robustness and to accurately capture the physical variance induced by thermal fluctuations and magnon scattering, all finite temperature continuous field switching results (e.g., at 4 K and 10 K) were averaged over an ensemble of 100 independent simulations, each initialized with a different random seed. The reported switching times and fields at these temperatures represent the mean values of these statistical distributions.

### 4.6 Determination of the Critical Static Field by the Bisection Method

To identify the minimum static magnetic field required to induce magnetization switching in CrSBr magnets, we employed the bisection method. The use of a static field assumes the presence of a threshold field strength above which a switching reliably occurs within the designated time frame. The procedure began with an upper bound sufficiently large to ensure switching and a lower bound considered insufficient for spin reversal. At each iteration, the midpoint of these bounds was used as the test field in atomistic simulations. If a switching occurred, the upper bound was updated to the midpoint; otherwise, the lower bound was set to the midpoint. This process was repeated until the difference between the bounds fell below a predefined precision. The critical field was then taken as the final midpoint, with the associated error given by the difference between the final bounds.

## Author Contributions

M.H.B. and E.J.G.S. initiated and designed the project. M.H.B. performed the OCT simulations, prepared the figures and analysis under the supervision of E.J.G.S. P.C. performed the atomistic spin dynamic simulations. E.J.G.S. wrote the manuscript with an initial draft prepared by M.H.B. All authors contributed to this work, read the manuscript, discussed the results, and agreed on the content of the manuscript.

## Conflicts of Interest

The authors declare no conflicts of interests.

## Supporting information


**Supporting File**: adma73969‐sup‐0001‐SuppMat.pdf.

## Data Availability

All the data supporting the findings of this study are available within the paper, the Supplementary Information, and under reasonable request.
