# Supplementary material for: Optimal Control Drives Ultrafast and Energy‐Efficient Magnetization Switching in Van der Waals Magnets
Source: Adv Mater. 2026 Jul 14;38(59):e23059. doi: 10.1002/adma.202523059 (PMC13689918; doi:10.1002/adma.202523059)
Supplement: Supplementary file 1 [file ADMA-38-e23059-s001.pdf]

Supplementary information for "Optimal control drives ultrafast and energy-efficient magnetization switching in van der Waals magnets"

Mohammad H. Badarneh[1†], PeiYu Cai[1†], & Elton J. G. Santos[1,2,3,⋆]

[1] *Institute for Condensed Matter Physics and Complex Systems, School of Physics and Astronomy, The University of Edinburgh, Edinburgh EH9 3FD, UK*

[2] *Higgs Centre for Theoretical Physics, The University of Edinburgh, Edinburgh EH9 3FD, UK*

[3] *Donostia International Physics Center (DIPC), 20018 Donostia-San Sebastián, Spain*

Contents

1 Optimal control paths and pulses in vdW magnets **4**

2 Torque Dynamics During Optimal Control of Magnetization in CrSBr **7**

3 Thermal Variance of Conventional Switching in CrSBr **9**

4 Maximum field strength as a function of switching time in monolayer CrSBr **14**

† These authors contributed equally to this work.

*Corresponding author: esantos@ed.ac.uk

5 Minimum energy cost of switching vs lateral size **15**

6 Variation with the number of layers **15**

7 Magnetization configurations at peak energy along optimal control paths **18**

8 Minimum energy cost of skyrmion annihilation versus skyrmion size in monolayer Fe_3GaTe_2 **19**

9 Topological charge along the OCP and MEP in Fe_3GaTe_2 **20**

10 Material-specific parameters **22**

11 Midpoint scheme for finding optimal control paths **24**

12 Generating initial guess for the OCT and GNEB calculations. **25**

13 Comparison to Memory Technologies **26**

14 Data repository **29**

15 References 29

1 Optimal control paths and pulses in vdW magnets

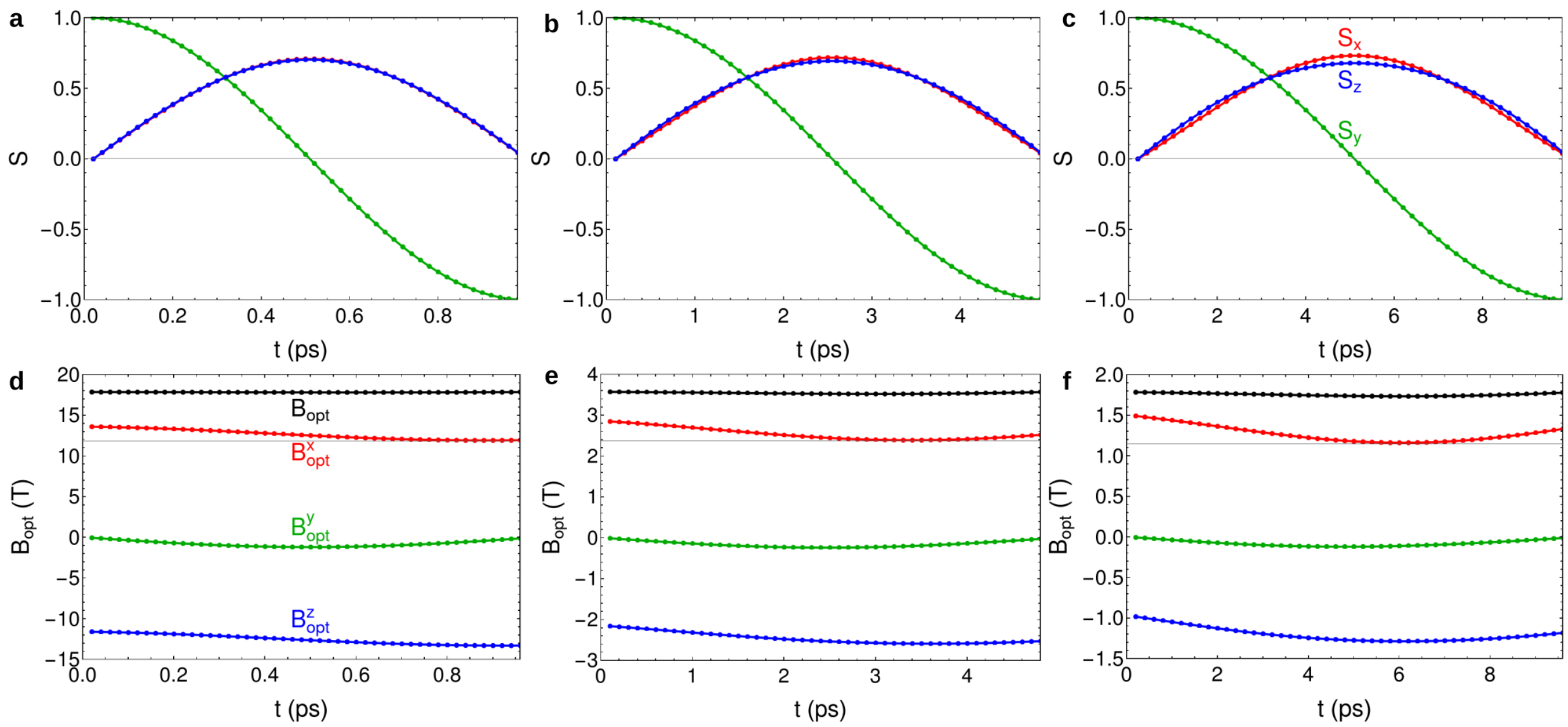

Supplementary Figure 1: **Optimal control of spin dynamics in monolayer CrSBr. a-c,** Optimal control path in CrSBr under optimally magnetic fields at zero temperature for different switching times $\tau = 1$ ps, $\tau = 5$ ps, and $\tau = 10$ ps, respectively. **d-f,** Corresponding optimal magnetic field components and total field magnitude as functions of time for the same switching durations shown in (**a-c**).

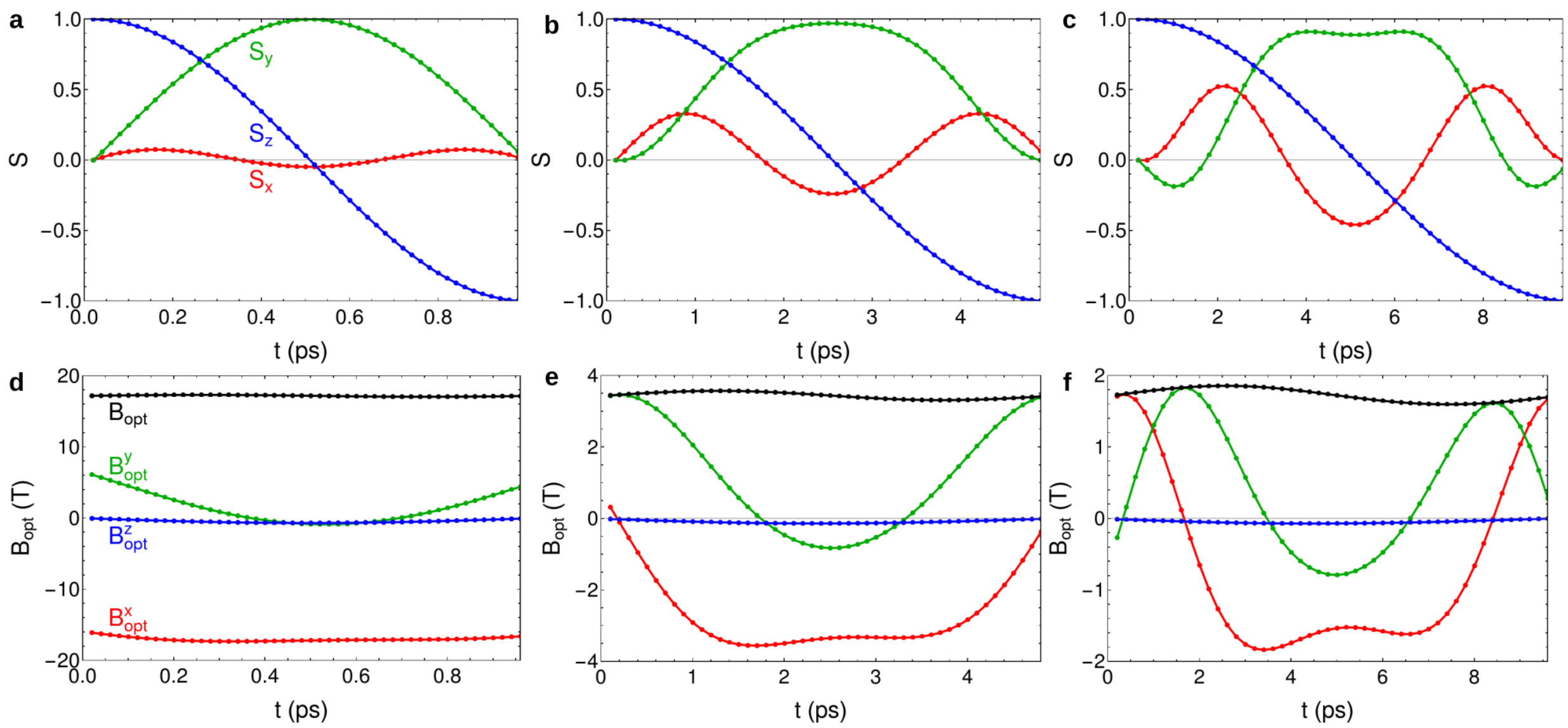

Supplementary Figure 2: **Optimal control of spin dynamics in monolayer Fe_3GaTe_2. a-c,** Optimal control path in Fe_3GaTe_2 under optimally magnetic fields at zero temperature for different switching times $\tau = 1$ ps, $\tau = 5$ ps, and $\tau = 10$ ps, respectively. **d-f,** Corresponding optimal magnetic field components and total field magnitude as functions of time for the same switching durations shown in (**a-c**).

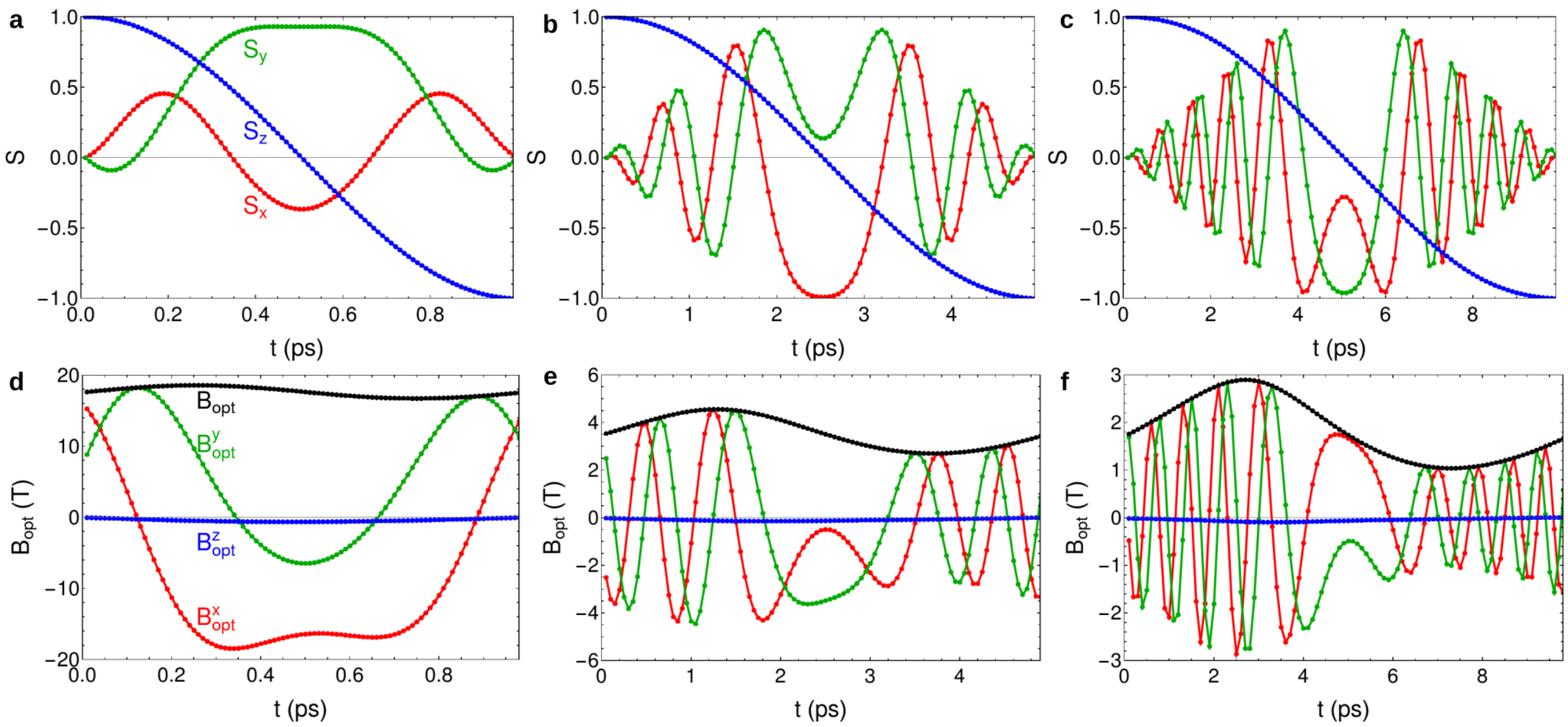

Supplementary Figure 3: **Optimal control of spin dynamics in monolayer Fe_3GeTe_2. a-c,** Optimal control path in Fe_3GeTe_2 under optimally magnetic fields at zero temperature for different switching times $\tau = 1$ ps, $\tau = 5$ ps, and $\tau = 10$ ps, respectively. **d-f,** Corresponding optimal magnetic field components and total field magnitude as functions of time for the same switching durations shown in (**a-c**).

2 Torque Dynamics During Optimal Control of Magnetization in CrSBr

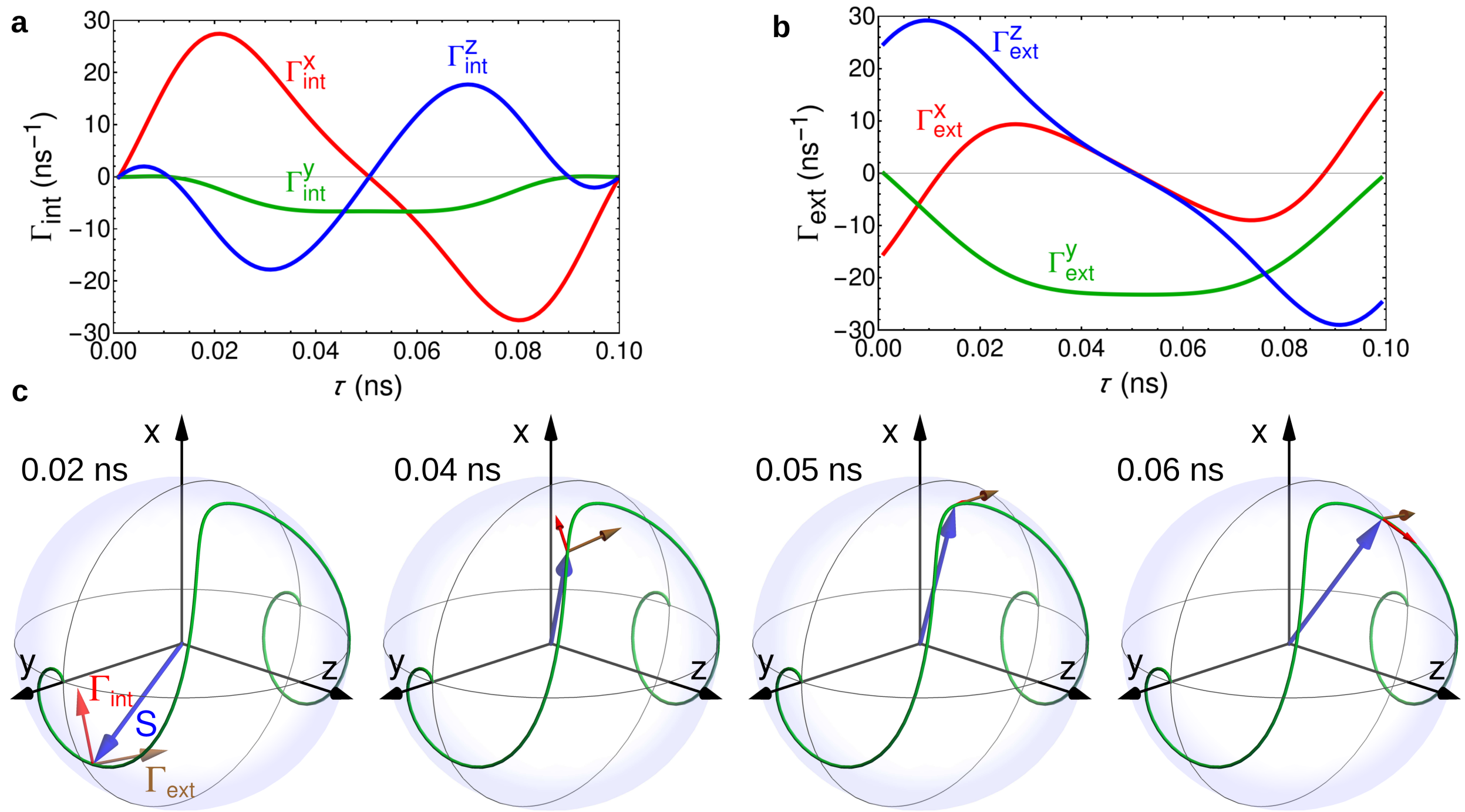

Supplementary Figure 4: **Internal and external torques along the optimal control path in CrSBr.** Time evolution of the internal torque $\mathbf{\Gamma}_{\text{int}}$ **(a)** and external torque $\mathbf{\Gamma}_{\text{ext}}$ **(b)** acting on the average magnetization **S** as a function of the switching time τ, evaluated along the optimal control path in a 5×5 nm^2 monolayer of CrSBr. Here, $\mathbf{\Gamma}_{\text{int}}$ originates from the intrinsic effective magnetic fields and damping torque, while $\mathbf{\Gamma}_{\text{ext}}$ arise from the time-dependent optimal control stimulus. The external torque drives the switching process and is tailored to ensure minimal energy dissipation. **(c)** Time-resolved snapshots of the optimal control path traced by the average magnetization vector **S** (green trajectory) on the unit sphere, shown at four representative time points (0.02, 0.04, 0.05, and 0.06 ns). At each time step, arrows denote the directions of the internal torque $\mathbf{\Gamma}_{\text{int}}$ (red), external torque $\mathbf{\Gamma}_{\text{ext}}$ (brown), and magnetization vector **S** (blue).

To quantify the strength of the time-dependent control magnetic field used in our OCT simulations, we define the instantaneous maximum field amplitude $B_{opt}^{max}(t) = \max(|B_{opt}|)$ for $t \in [0, \tau]$. This provides a clear, time-resolved measure of how the control is applied throughout the magnetization switching process. However, it is important to note that our optimization does not aim to minimize this quantity. The control objective is to minimize Joule heating which depends on the time-integrated power, not on peak field values (see *Methods* for details). As a result, transiently large values of $B_{opt}^{max}(t)$ may appear at specific times, but these arise naturally from the optimization, which balances energy cost with dynamical effectiveness. The external torque $\mathbf{\Gamma}_{\text{ext}}(t)$, resulting from the optimal pulse and shown in the Supplementary Fig. S4(**a-c**), reflects this behavior in CrSBr. In Fig. S4(**a**) the internal torque $\mathbf{\Gamma}_{\text{int}}(t)$ vanishes at both $\tau = 0$ and $\tau = 0.1$ ns, consistent with the system starting and ending in local energy minima at $\pm y$. During the first half of the reversal process, $\mathbf{\Gamma}_{\text{int}}(t)$ counteracts the reversal due to the damping toque, while in the second half it assists the reversal as the system approaches the target state. In Fig. S4(**b**), $\mathbf{\Gamma}_{\text{ext}}(t)$ exhibits non-zero components in the x- and z-directions. These components push the system out of the minimum at $+y$ and toward the minimum at $-y$ at $\tau = 0$ and $\tau = 0.1$ ns, respectively. Fig. S4(**c**) shows the time-resolved trajectory of the average magnetization vector $\mathbf{S}$ (green path) on the unit sphere, illustrated at four representative times (0.02, 0.04, 0.05, and 0.06 ns). At each time step, arrows indicate the directions of $\mathbf{\Gamma}_{\text{int}}$ (red), $\mathbf{\Gamma}_{\text{ext}}$ (brown), and $\mathbf{S}$ (blue). These visualizations highlight the interplay between internal and external torques during the switching mechanism in CrSBr and the crucial role of optimal control in guiding the system along the desired low-energy transition path. Building on this microscopic understanding of the torque dynamics and control field behavior, we

next evaluate the efficiency of the OCT approach by comparing it with conventional switching protocols across different switching times.

3 Thermal Variance of Conventional Switching in CrSBr

Here we further explored finite-temperature effects on the conventional switching (Supplementary Figure 5). To ensure the analysis of these thermal effects is statistically robust and free from single-run stochastic artifacts, we performed extensive statistical sampling using 100 independent simulations with different random seeds for each applied field and temperature (0 K, 4 K, 10 K). The ensemble-averaged results (Supplementary Figure 5**a**) confirm that the previously observed wide variance at higher temperatures stems from intrinsic physical crossovers between distinct dynamical regimes rather than computational artifacts. In the sub-nanosecond regime, conventional switching relies on deterministic coherent rotation. A successful reversal requires the magnetization to overcome the anisotropy barrier at the equatorial plane and subsequently dissipate sufficient kinetic and Zeeman energy via the intrinsic damping mechanism to become trapped in the target hemisphere. However, the Larmor precession frequency is directly proportional to the applied magnetic field. Comparing the time-resolved magnetization trajectories under applied fields of 0.6 T and 0.9 T illustrate this delicate balance (Supplementary Figure 5**b**). While the 0.6 T field allows the magnetization to damp into the reversed state after an initial precessional bounce ($\sim$100 ps), the elevated 0.9 T field drives the Larmor frequency to higher values. Consequently, the magnetization sweeps past the equator too rapidly. Because the spin spends an insufficient amount of time near the equator, the damping torque cannot dissipate the necessary energy. This dynamic over-

shoot forces the magnetization to rotate back, initiating high frequency precessional ringing. As highlighted by the red arrow at $\sim$170 ps in the 0.9 T trajectory (Supplementary Figure 5**b**), the system transiently rebounds rather than cleanly settling into the energy minimum, thus increasing the total switching time. This delicate interplay between the field-driven Larmor frequency, the transit time near the equator, and the fixed damping rate produces the sharp, non-monotonic oscillatory boundaries observed in the B_{conv} switching at 0 K below 5 T (Supplementary Figure 5**a**).

When finite temperatures are introduced (4 K, 10 K), our 100-seed ensemble averages reveal distinct regimes dictated by the applied field strength. In Supplementary Figure 5**a**, the red dashed line at 13 ps is included to indicate the ultrafast timescale presented in Figure 1i of the main text. However, the pure field-driven switching mechanism extends well beyond this 13-ps marker. Specifically, for applied fields above $\sim$5 T, the reversal process is entirely dominated by the initial driving torque, which is characterized by a strict inverse monotonic relationship between the applied field value and the required switching time. In this high-field regime, higher temperatures such as 10 K induce larger initial random angular deviations of the atomic spins from their equilibrium easy axis. This thermally broadened initial angle maximizes the cross product between the magnetization and the applied massive field, dramatically accelerating the onset of the initial torque. Consequently, the 10 K system switches faster and requires less field than the 4 K or 0 K systems, consistent with the trend shown in Figure 1**i**.

Conversely, for applied fields below 5 T, the switching behavior transitions into a regime governed by precessional phase matching and stochastic thermal activation. At 10 K, the height-

ened thermal energy induces stronger magnon scattering and thermal dephasing, which disrupts the uniform precessional mode. However, our statistical data demonstrates that the additional thermal energy provides a stronger activation assist over the static anisotropy barrier, which ultimately benefits the reversal process more than the scattering disrupts it. As a result, the ensemble-averaged switching time at 10 K remains lower than that at 4 K. The critical consequence of the thermal dephasing is not an increase in the average switching time, but rather a massive increase in the statistical variance of the reversal trajectories. Because the individual spins lose synchronization, the exact trajectory and stabilization time become highly unpredictable. This is clearly indicated by the exceptionally wide horizontal error bars representing the standard deviation for the 10 K data at low fields ($<$1 T) (Supplementary Figure 5**a**). To provide a visual proof of this dephasing mechanism, Supplementary Figure 5**c** five individual magnetization trajectories evaluated at a fixed field of 0.6 T. While the simulations at 0 K yield a single deterministic path, the 10 K trajectories spread into a wide distribution of distinct, noisy trajectories, vividly demonstrating how thermal energy disrupts the uniform precessional mode. The stark contrast between the deterministic precessional path at 0 K and the widely dispersed paths at 10 K illustrates how thermal noise shatters phase coherence, leading to the wide standard deviations observed in the low field ensemble averages.

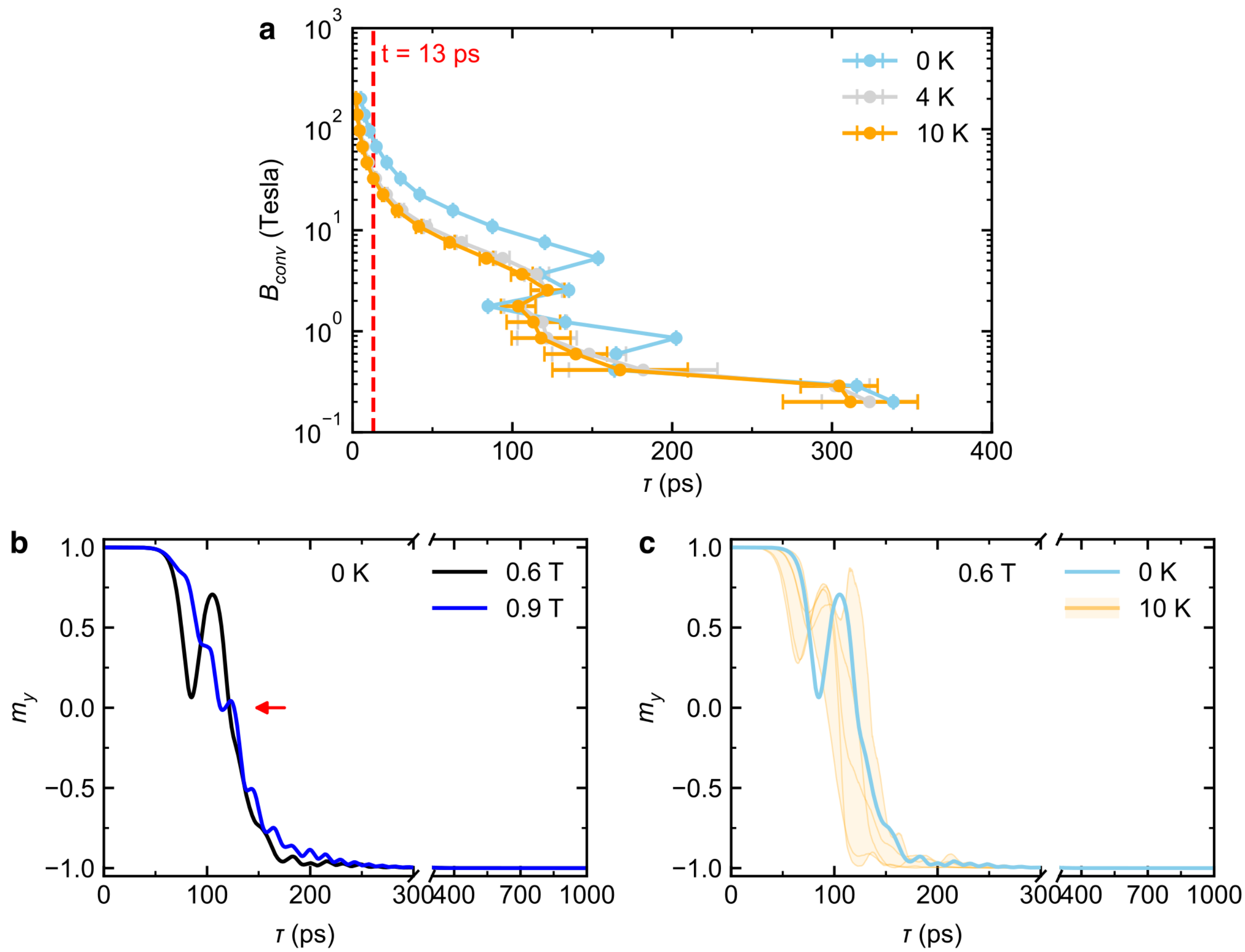

Supplementary Figure 5: **Statistical temperature dependence and zero-temperature magnetization trajectories in CrSBr. a,** Ensemble-averaged conventional switching phase diagrams derived from 100 independent statistical seeds at 0 K, 4 K, and 10 K. The error bars indicate the standard deviation of the switching times, highlighting the increased statistical variance at higher temperatures in the low field precessional regime. **b,** Time-resolved zero temperature longitudinal magnetization trajectories which contrasted an efficient precessional reversal at 0.6 T with a severe dynamic overshoot and subsequent ringing at 0.9 T. **c,** Individual time-resolved longitudinal magnetization trajectories at an applied field of 0.6 T, comparing the deterministic precessional reversal at 0 K with the widely dispersed paths at 10 K caused by thermal effects averaged across 5 random seeds.

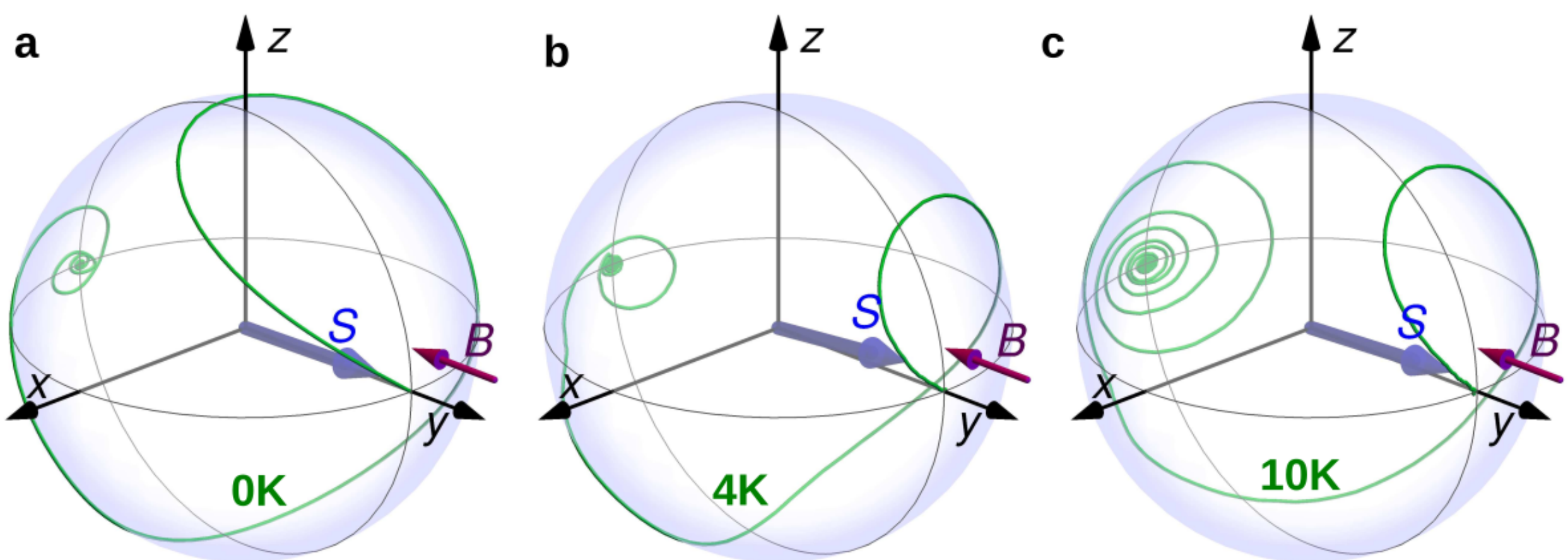

Supplementary Figure 6: **Temperature-dependent magnetization dynamics in CrSBr.** Averaged magnetization dynamics in CrSBr induced by a static magnetic field of 0.5 Tesla at different temperatures 0 K (**a**), 4 K (**b**), and 10 K (**c**).

4 Maximum field strength as a function of switching time in monolayer CrSBr

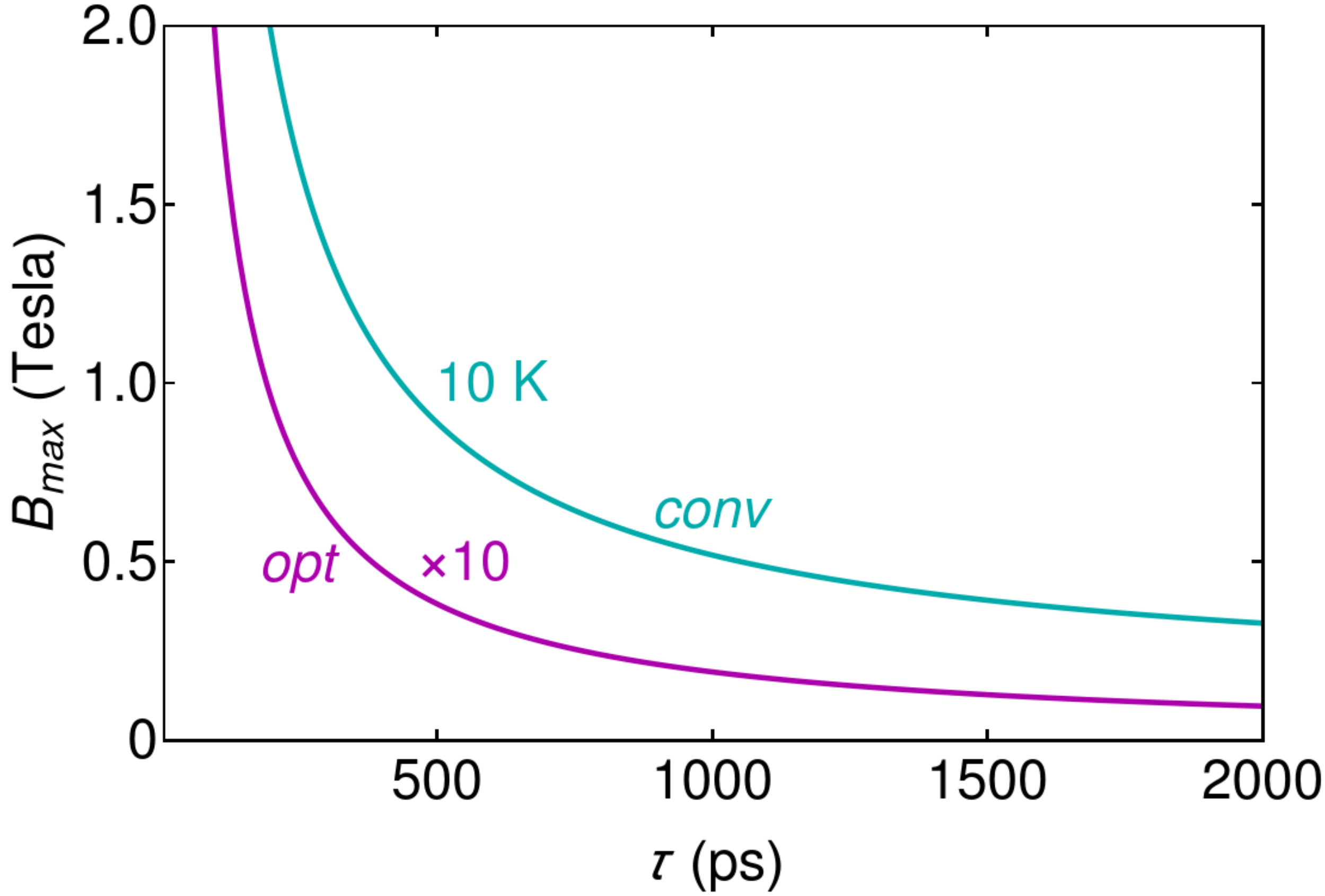

Supplementary Figure 7: **Maximum field strength vs switching time for magnetization reversal in monolayer CrSBr.** Dependence of the maximum magnetic field strength B_{max} on the total switching time τ for magnetization reversal in a 50×50 nm^2 monolayer of CrSBr. The system is initially in a uniform ferromagnetic state with magnetization aligned along the $+y$ direction. A magnetic field is applied along the $+y$ direction to reverse the magnetization. The cyan curve labeled corresponds to a conventional protocol using a static field, evaluated at 10 K. The magenta curve shows the result of an optimal control protocol where the time-dependent field is engineered to minimize the required maximum field strength while achieving complete reversal.

5 Minimum energy cost of switching vs lateral size

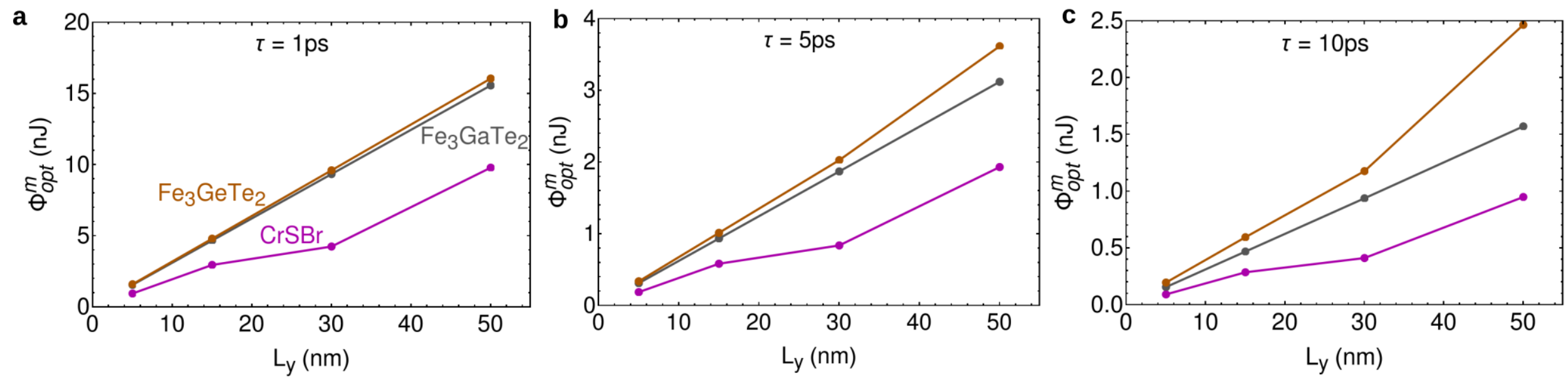

Supplementary Figure 8: **Energy cost of magnetization switching in monolayer vdW magnets at different lateral sizes and switching times** Minimum energy cost of magnetization switching as a function of lateral size in vdW magnets for different switching times **a** 1ps, **b** 5ps, and **c** 10ps.

6 Variation with the number of layers

To examine the effect of number of layers on the switching costs, we carried out additional OCT simulations for bilayer (2L) and trilayer (3L) CrSBr as a representative example and plotted them together with the monolayer (1L) results for a comparison. Supplementary Figure 9**a** shows that the OCT switching cost energy Φ_m increases with the number of layers, which is an expected behavior since more spins must be reoriented. However, the maximum switching field amplitude $B_\mathrm{opt}^\mathrm{max}$ remains almost constant (Supplementary Figure 9**b-c**) with small increments (~ 0.01 T) at a determined switching time (50 ps). We understand this result given that in the OCT approach the magnetic field pulse is optimized so that all layers reverse their magnetization within the predefined switching time. As a result, the peak field is mainly set by the most demanding layer, namely the one that requires the largest external torque (from the external magnetic field) at a given time to overcome the internal torque (which arises from the damping torque and the effective field). In 2L

and 3L CrSBr, the coupling between adjacent layers is relatively weak, so the layers behave almost independently under the applied field. Because all layers have the same structure and magnetic properties, adding more layers does not significantly change the condition that determines the required peak field.

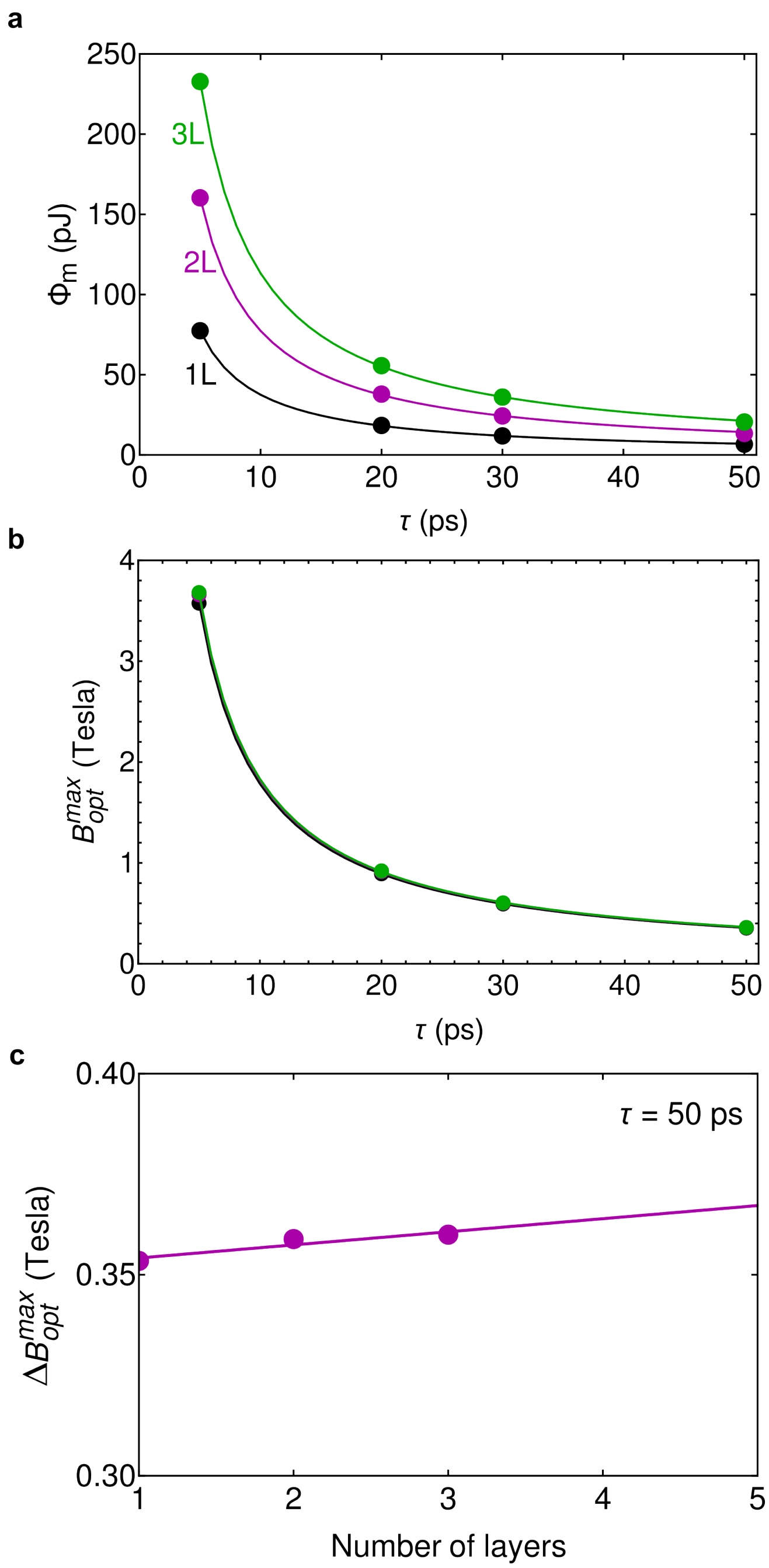

Supplementary Figure 9: **OCT simulations for different number of CrSBr layers (1L, 2L, 3L)**. **a,** Minimum energy cost Φ_{m}(pJ) versus switching time τ(ps) for 1L-3L. **b-c,** Maximum optimum filed $B_{\mathrm{opt}}^{\mathrm{max}}$ as a function of τ with its differential increment across the layers at $\tau = 50$ ps, respectively. The lateral system size is 10×10 nm^2.

7 Magnetization configurations at peak energy along optimal control paths

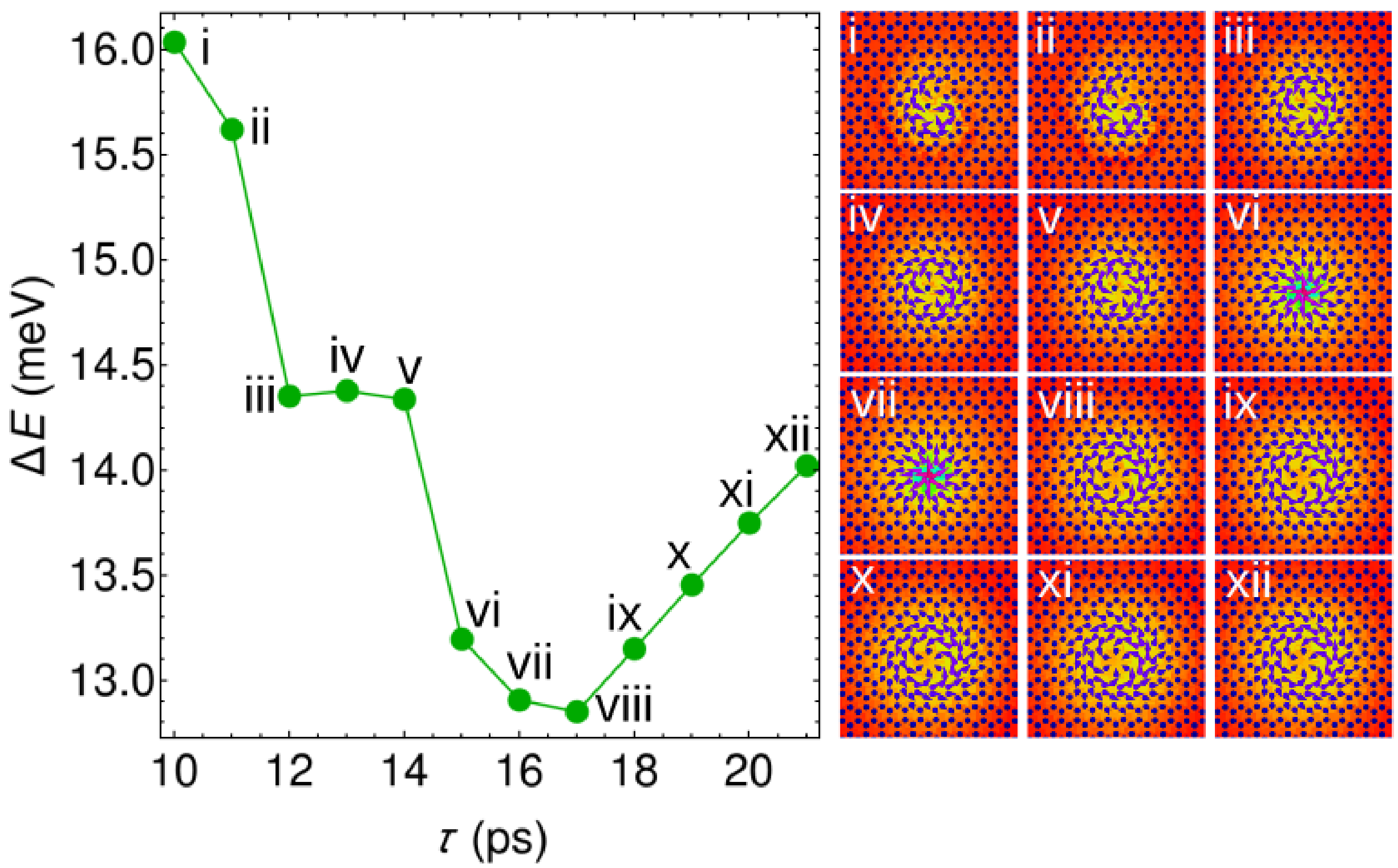

Supplementary Figure 10: **Magnetization configurations at peak energy points along optimal control paths. a,** The energy difference between the initial state and the highest energy point along the optimal control paths shown in Fig. 3 (**a**) in the main text for a given switching time τ. **b,** The magnetization configuration corresponding to the peak energy point.

8 Minimum energy cost of skyrmion annihilation versus skyrmion size in monolayer Fe_3GaTe_2

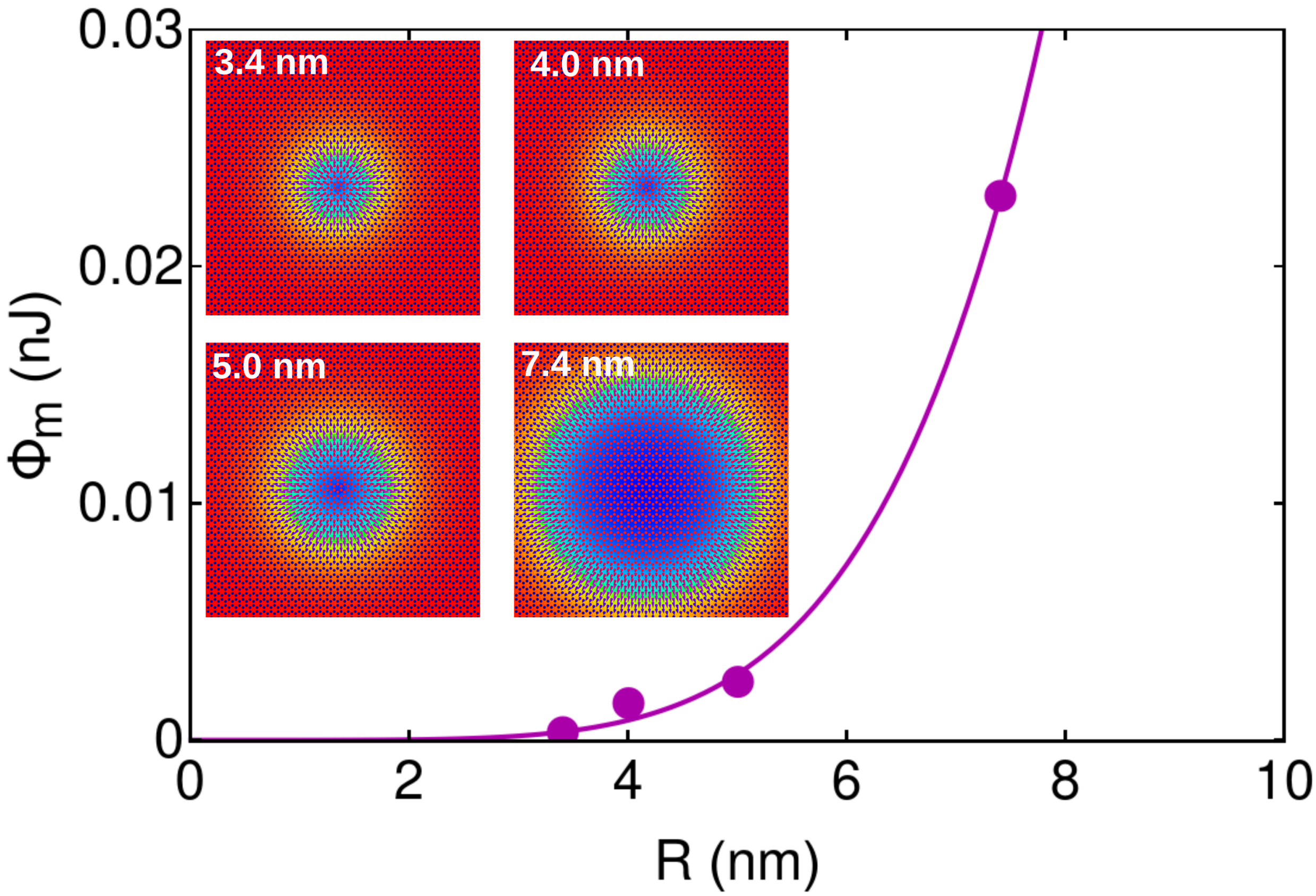

Supplementary Figure 11: **Minimum energy cost of skyrmion annihilation in monolayer Fe_3GaTe_2.** Symbols denote OCT data, and the solid curve is a fit. Insets show magnetic configurations for different radii (3.4, 4.0, 5.0 and 7.4 nm). The system size is 50×50 nm^2.

9 Topological charge along the OCP and MEP in Fe_3GaTe_2

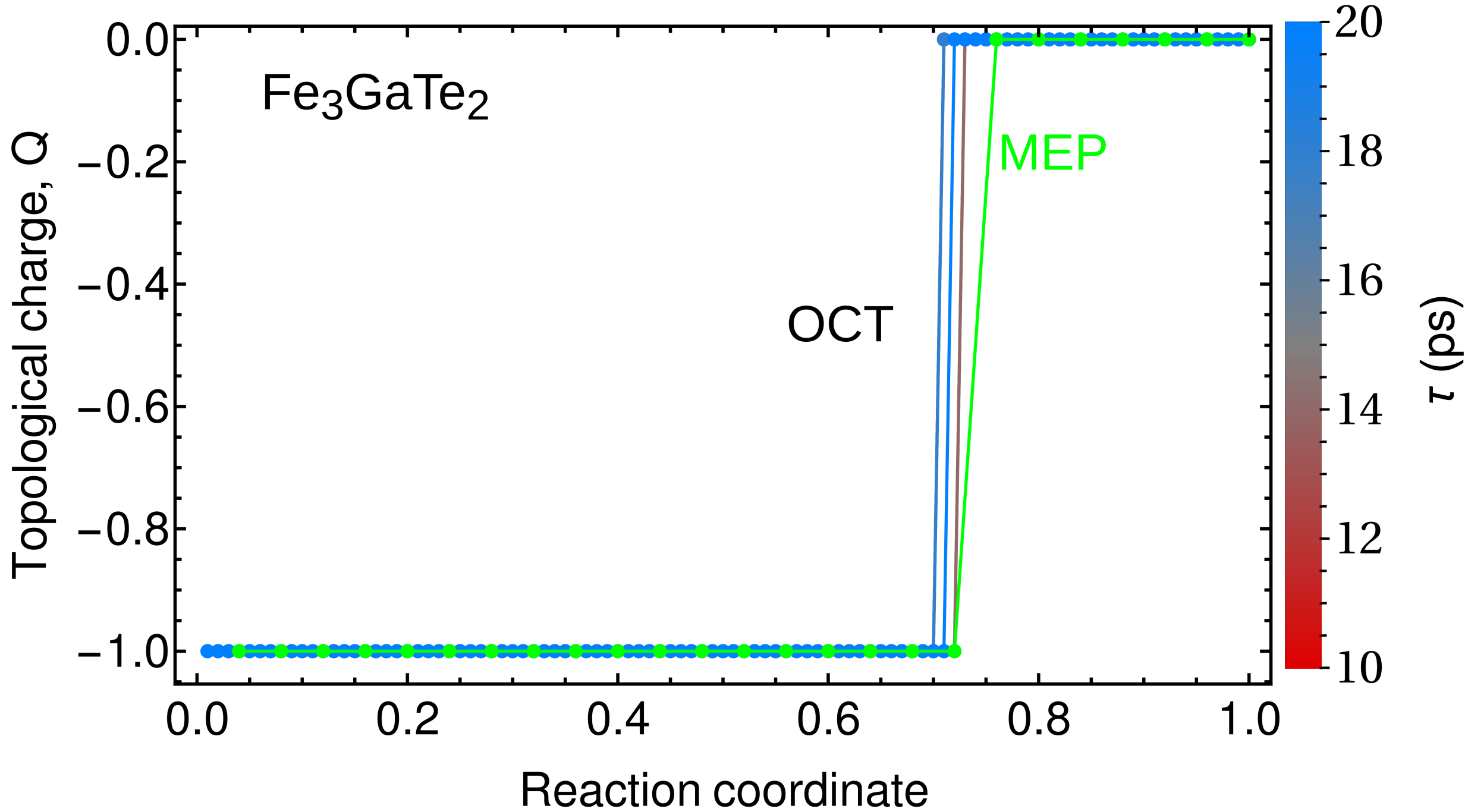

Supplementary Figure 12: **Topological charge Q along the MEP and OCP for skyrmion annihilation in Fe_3GaTe_2.** The reaction coordinate is defined as the normalized displacement along the minimum energy path (MEP, green line) and the normalized time along the optimal control path (OCP, color-coded by the switching time τ). The topological charge exhibits a sharp transition from Q = −1 (skyrmion state) to Q = 0 (ferromagnetic state) near the saddle point along both pathways, indicating a topological change associated with the annihilation process.

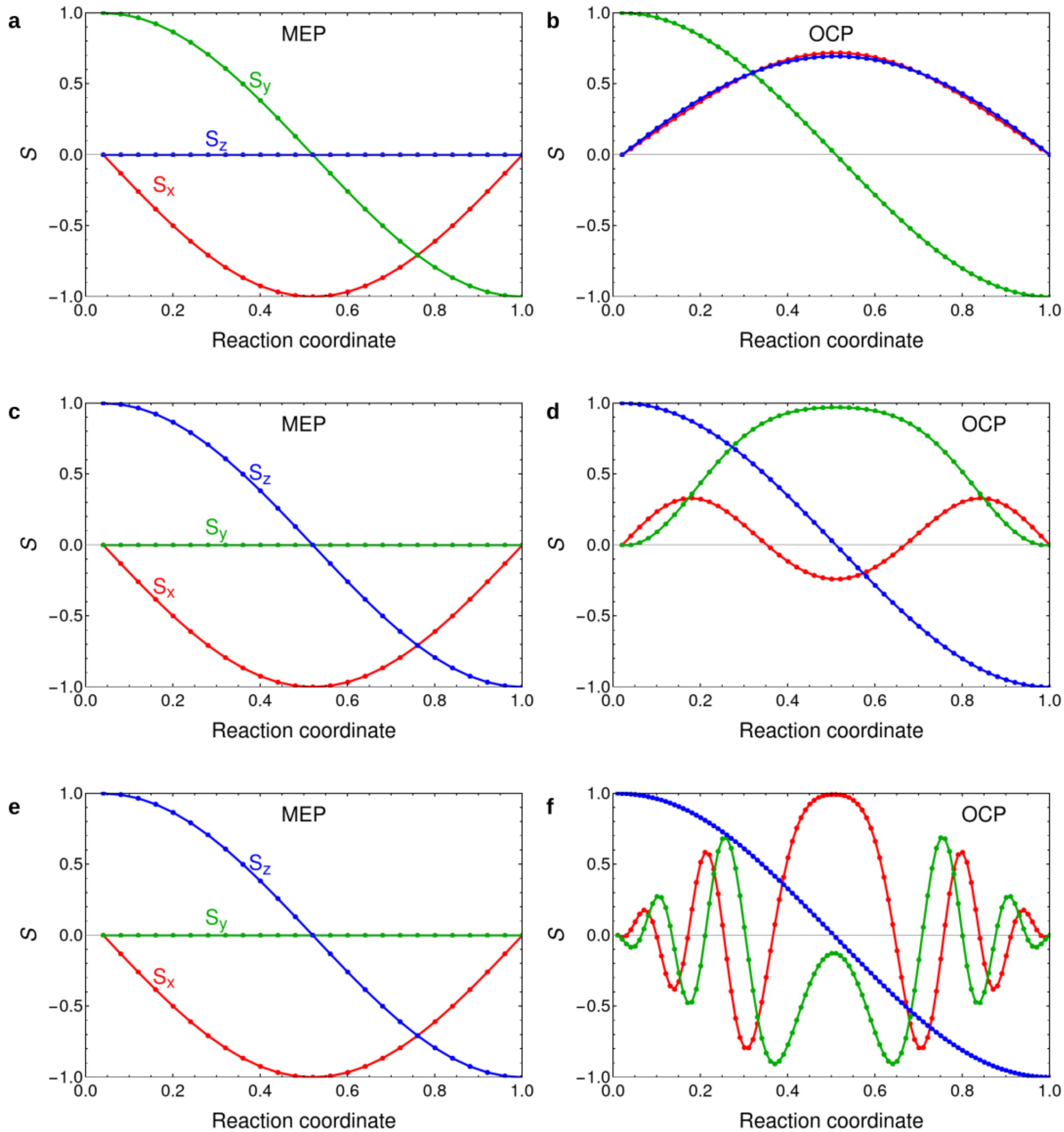

Supplementary Figure 13: **Spin-component trajectories along MEP and OCP in CrSBr, Fe_3GaTe_2, and Fe_3GeTe_2. a-b,** Evolution of the spin components (S_x, S_y, S_z) versus reaction coordinate during magnetization reversal in a $50 \times 50\,\mathrm{nm}^2$ system size for CrSBr. **c-d,** Similar as **a-b** but for Fe_3GaTe_2. **e-f,** Similar as **a-b** but for Fe_3GeTe_2. The reaction coordinate is defined as the normalized displacement along the minimum energy path (MEP) and the normalized time along the optimal control path (OCP).

10 Material-specific parameters

We investigated three layered van der Waals magnets—Fe_3GaTe_2, Fe_3GeTe_2, and CrSBr—each exhibiting distinct lattice geometries, exchange interactions, DMI, anisotropy strengths, etc. The following material-specific parameters are used in the simulations.

CrSBr. A monolayer is considered with in-plane unit cell parameters obtained by DFT calculations $a = 3.545$ Å and $b = 4.733$ Å [1], consistent with experimentally reported values [2]. The atomic magnetic moment μ_s was included in the model, with a value of $\mu_s = 2.88\ \mu_B$ assigned according to bulk measurements [1], where μ_B is the Bohr magneton. The value of the damping parameter α is 0.066 [3]. The exchange parameters J_{ij}, first term in Eq. (15) in the main text, are obtained from Table (2) as shown in ref [1]. Due to the broken inversion symmetry between the interacting Cr-based atoms, the contribution of DMI, second term in Eq. (15) in the main text, was included in the simulations with DMI unit vectors parallel to the a-th and b-th axes, with DMI constants given by $D_1 = 0.07$ and $D_3 = 0.18$ meV, respectively [1]. Moreover, the system exhibits biaxial anisotropy axes, third term in Eq. (15) in the main text, which govern the intermediate a- and easy b-th axes of the system, with anisotropy constants $K_a = 8.06\mu$eV and $K_a = 31.53\mu$eV [4,5].

Fe_3XTe_2 (X: Ga, Ge). Considering the chemical and structural similarity between Fe_3GaTe_2 and Fe_3GeTe_2, which retains ferromagnetism and strong perpendicular anisotopy down to the monolayer, we consider in-plane unit cell parameters obtained by first-principles calculations $a = b = 3.94694$ Å [6,7]. To model the magnetic properties of these materials, we included two equivalent Fe atoms and one inequivalent Fe atom per unit cell [7]. For Fe_3GeTe_2, we assign mag-

netic moments of 1.95 μ_B to the equivalent Fe atoms and 1.56 μ_B to the inequivalent one [8]. In Fe_3GaTe_2, the corresponding values are 2.01 μ_B for the equivalent sites and 1.31 μ_B for the inequivalent one [7]. The value of the damping parameter α is 0.039 for Fe_3GaTe_2 and 0.035 for Fe_3GeTe_2 [9]. The exchange parameters J_{ij} between the equivalent and inequivalent Fe atoms are summarized in Ref. [7]. Moreover, the DMI occurs between nearest neighbor equivalent atoms, with the vectors primarily pointing perpendicular to the FGT film and alternating in sign for adjacent neighbors. However, because of mirror symmetry, the DMI contributions from equivalent atoms cancel out. Consequently, only the DMI between equivalent and inequivalent atoms is considered. Note that the second-largest contribution comes from nearest-neighbor interactions between equivalent and inequivalent atoms, while all other DMI pairs are at least an order of magnitude smaller[10]. The value of the DMI constant is considered to be 0.1 meV for both Fe_3GaTe_2 and Fe_3GeTe_2, respectively. Both Fe_3GaTe_2 and Fe_3GeTe_2 exhibit a strong perpendicular anisotopry with spins aligned along the c axis, with anisotropy constants $K_1 = 0.36$ meV and $K_1 = 2.83$ meV for Fe_3GaTe_2 and Fe_3GeTe_2, respectively [7,11].

11 Midpoint scheme for finding optimal control paths

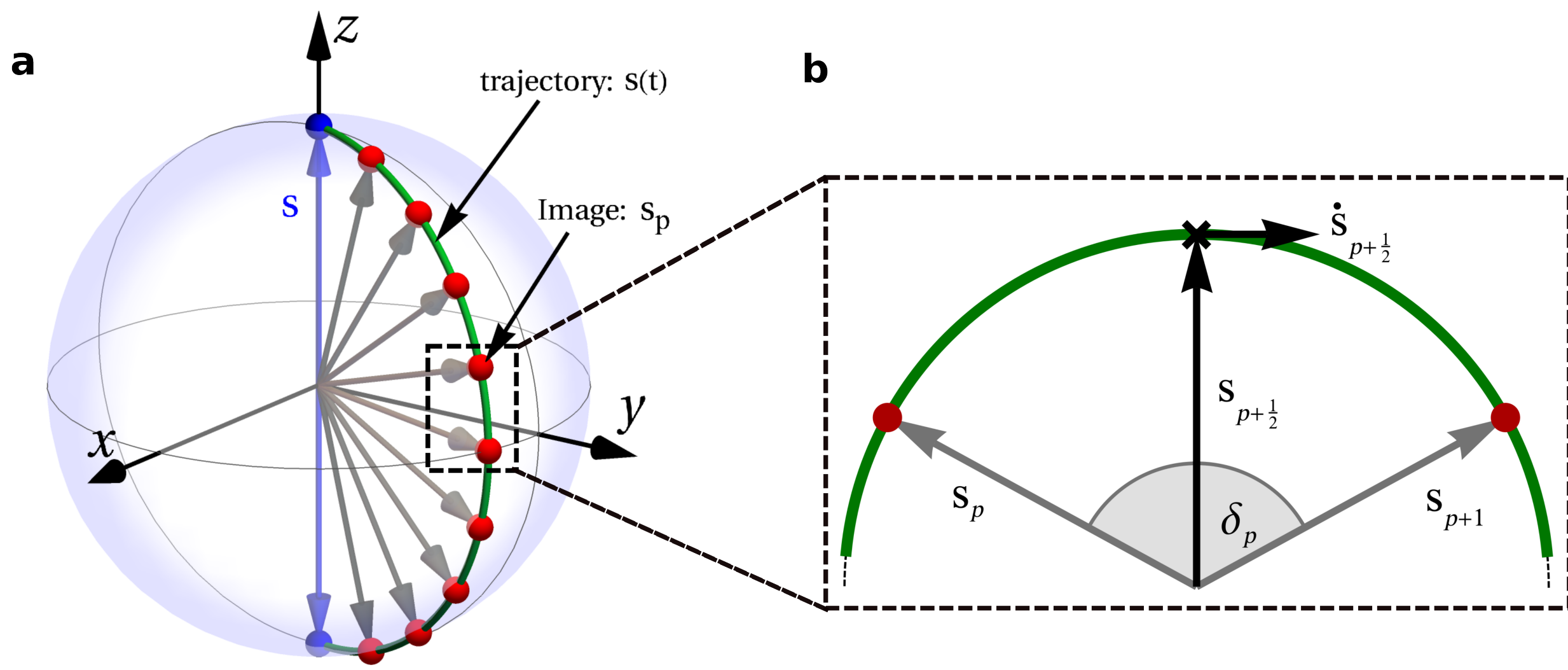

Supplementary Figure 14: **Midpoint-based discretization scheme for finding optimal control path. a,** Illustration of a trajectory, indicated by the green line, connecting the initial and final stable states of a macrospin pointing along the unit vector $\vec{s}$. The initial and final stable states are marked with blue points. Along this trajectory, each discrete point, represented by a red point, corresponds to a state of the system in the configuration space and is referred to as an image denoted by $\vec{s}_p = \vec{s}(t_p)$. These red points signify movable images that can be repositioned in the configuration space during the optimization process. **b,** Illustration of the midpoint scheme used in the numerical method for finding OCPs. Two images $\vec{s}_p$ and $\vec{s}_{p+1}$ are connected by a geodesic path in the configuration space. The position $\vec{s}_{p+\frac{1}{2}}$ and the velocity $\dot{\vec{s}}_{p+\frac{1}{2}}$ at the midpoint of the path are defined by $\vec{s}_p$ and $\vec{s}_{p+1}$, and the angle δ_p between them.

12 Generating initial guess for the OCT and GNEB calculations.

Some initial arrangement of the images is needed to start an OCP or GNEB calculations. This can be generated, for example, by placing the images uniformly along the shortest-distance path between the initial state $\mathscr{M}_0$ and the final state $\mathscr{M}_{Q+1}$ of the transition. In particular, generating an intermediate movable images Q along the short geodesic path can be achieved by rotating the magnetic moments from $\mathscr{M}_0$ to $\mathscr{M}_{Q+1}$ using Rodrigues' formula [12,13] as follows:

$$\vec{s}_{q,i} = \vec{s}_{0,i} \cos\theta_{q,i} + (\vec{k}_i \times \vec{s}_{0,i}) \sin\theta_{q,i}, \tag{1}$$

with $q = (1, 2, \ldots, Q)$. Here, $\vec{s}_{0,i}$ is the i-th magnetic unit vector in the zeroth image, while $\vec{s}_{q,i}$ is the ith magnetic unit vector in the qth image. $\theta_{q,i} = (q-1)\theta_i/Q$ is rotation angle with θ_i being an angle between the vectors $\vec{s}_{0,i}$ and $\vec{s}_{Q+1,i}$ belonging to the initial and final fixed images, respectively. The unit vector $\vec{k}_i$ describing an axis of rotation is defined as

$$\vec{k}_i \quad = \quad \frac{(\vec{s}_{0,i} \times \vec{s}_{Q+1,i})}{|(\vec{s}_{0,i} \times \vec{s}_{Q+1,i})|}. \tag{2}$$

It is also recommended to add small random noise to the initial path to avoid convergence on maxima or saddle points of $\Phi(\mathbf{s})$ due to possible symmetries in the system. A local minimization of $\Phi(\mathbf{s})$ will most likely converge to the OCP closest to the path generated initially. If multiple OCPs are present between the initial and final states, several initial estimates need to be produced to enable convergence on the various solutions.

13 Comparison to Memory Technologies

Tables 1-6 summarize the write speed and energy per bit for various memory technologies, including static random access memory (SRAM), spin-transfer torque magnetoresistive random access memory (SOT-MRAM), spin-orbit torque magnetoresistive random access memory (STT-MRAM), and voltage-gate-assisted spin-orbit torque magnetoresistive random access memory (VGSOT-MRAM). The data highlight the trade-offs between switching speed and energy efficiency across different memory devices.

Supplementary Table 1: Write speed and energy per bit for the SRAM technology.

**Write Speed (ns)**	**Energy/bit (fJ)**	**Reference**
0.25	6.0×10^2	[14]
0.8	1.11×10^4	[15]
1.0	1.0×10^2	[16]
5.0	7.23×10^0	[17]
6.05	1.255×10^4	[18]

Supplementary Table 2: Write speed and energy per bit for the DRAM technology.

**Write Speed (ns)**	**Energy/bit (fJ)**	**Reference**
12	1.11×10^5	[19]
15	3.90×10^2	[20]
15	7.00×10^4	[19]
20	1.00×10^3	[16]

Supplementary Table 3: Write speed and energy per bit for SOT-MRAM technology.

**Write Speed (ns)**	**Energy/bit (fJ)**	**Reference**
0.2	2.0×10^3	[21]
0.27	4.0×10^2	[22]
0.4	1.4×10^3	[21]
0.5	4.5×10^2	[23]
1.0	1.0×10^3	[21]
7.609	1.567×10^5	[24]
9.883	1.23×10^5	[24]
10.0	1.504×10^3	[25]
12.114	1.506×10^5	[24]

Supplementary Table 4: Write speed and energy per bit for the STT-MRAM technology.

Write Speed (ns)	Energy/bit (fJ)	Reference
0.275	1.2×10^2	[26]
0.618	5.4×10^1	[27]
1.0	3.0×10^2	[28]
8.7	3.01×10^2	[29]
10.0	2.737×10^3	[25]
13.0	4.58×10^2	[29]

Supplementary Table 5: Write speed and energy per bit for the VGSOT-MRAM technology.

Write Speed (ns)	Energy/bit (fJ)	Reference
0.3	6.0×10^1	[30]
0.4	3.0×10^1	[31]
1.0	7.5×10^1	[32]
1.0	5.0×10^1	[21]

Supplementary Table 6: Energy required for skyrmion nucleation

Write Speed (ns)	Energy/bit (fJ)	Reference
4	5.0×10^5	[33]
10	1.0×10^2	[34]
10	3.0×10^4	[35]

14 Data repository

The dataset included in the main text can be found at the following repository.

15 References

1. X. Bo, F. Li, X. Xu, X. Wan, and Y. Pu, "Calculated magnetic exchange interactions in the van der Waals layered magnet CrSBr," *New Journal of Physics*, vol. 25, no. 1, p. 013026, 2023.

2. E. J. Telford, A. H. Dismukes, K. Lee, M. Cheng, A. Wieteska, A. K. Bartholomew, Y.-S. Chen, X. Xu, A. N. Pasupathy, X. Zhu, *et al.*, "Layered antiferromagnetism induces large negative magnetoresistance in the van der Waals semiconductor CrSBr," *Advanced Materials*, vol. 32, no. 37, p. 2003240, 2020.

3. T. M. J. Cham, D. G. Chica, K. Watanabe, T. Taniguchi, X. Roy, Y. K. Luo, and D. C. Ralph, "Spin-filter tunneling detection of antiferromagnetic resonance with electrically-tunable damping," *arXiv preprint arXiv:2407.09462*, 2024.

4. H. Wang, J. Qi, and X. Qian, "Electrically tunable high curie temperature two-dimensional ferromagnetism in van der Waals layered crystals," *Applied Physics Letters*, vol. 117, no. 8, 2020.

5. Y. Zur, A. Noah, C. Boix-Constant, S. Mañas-Valero, N. Fridman, R. Rama-Eiroa, M. E. Huber, E. J. Santos, E. Coronado, and Y. Anahory, "Magnetic imaging and domain nucleation in CrSBr down to the 2D limit," *Advanced Materials*, vol. 35, no. 47, p. 2307195, 2023.

6. C. Xu, X. Li, P. Chen, Y. Zhang, H. Xiang, and L. Bellaiche, "Assembling diverse skyrmionic phases in Fe_3GeTe_2 monolayers," *Advanced Materials*, vol. 34, no. 12, p. 2107779, 2022.

7. A. M. Ruiz, D. L. Esteras, D. López-Alcalá, and J. J. Baldoví, "On the origin of the above-room-temperature magnetism in the 2D van der Waals ferromagnet Fe_3GaTe_2," *Nano Letters*, vol. 24, no. 26, pp. 7886–7894, 2024.

8. V. Y. Verchenko, A. A. Tsirlin, A. V. Sobolev, I. A. Presniakov, and A. V. Shevelkov, "Ferromagnetic order, strong magnetocrystalline anisotropy, and magnetocaloric effect in the layered telluride $Fe_3 - \delta GeTe_2$," *Inorganic chemistry*, vol. 54, no. 17, pp. 8598–8607, 2015.

9. J. Zhang, Z. Wang, Z. Li, T. Li, S. Liu, J. Zhang, R.-J. Zhang, Q. Jin, Z. Shi, Y. Liu, *et al.*, "Sub-THz high spin precession frequency in van der Waals ferromagnet Fe_3GaTe_2," *Nano Letters*, vol. 24, no. 39, pp. 12204–12210, 2024.

10. H.-H. Yang, N. Bansal, P. Rüßmann, M. Hoffmann, L. Zhang, D. Go, Q. Li, A.-A. Haghighirad, K. Sen, S. Blügel, *et al.*, "Magnetic domain walls of the van der Waals material Fe_3GeTe_2," *2D Materials*, vol. 9, no. 2, p. 025022, 2022.

11. Q. Liu, J. Xing, Z. Jiang, Y. Guo, X. Jiang, Y. Qi, and J. Zhao, "Layer-dependent magnetic phase diagram in Fe_nGeTe_2 ($3 \leq n \leq 7$) ultrathin films," *Communications Physics*, vol. 5, no. 1, p. 140, 2022.

12. R. M. Murray, Z. Li, and S. S. Sastry, *A mathematical introduction to robotic manipulation*. CRC press, 2017.

13. P. F. Bessarab, V. M. Uzdin, and H. Jónsson, "Method for finding mechanism and activation energy of magnetic transitions, applied to skyrmion and antivortex annihilation," *Computer Physics Communications*, vol. 196, pp. 335–347, 2015.

14. M. A.-A. Kaiser, S. Sunder, C. Mathew, M. Rakowski, A. P. Jacob, and A. R. Jaiswal, "Design of energy-efficient cross-coupled differential photonic-sram (PSRAM) bitcell for high-speed on-chip photonic memory and compute systems," *arXiv preprint arXiv:2503.19544*, 2025.

15. J. Zhu, N. Bai, and J. Wu, "A low active and leakage power SRAM using a read and write divided and BIST programmable timing control circuit," *Microelectronics Journal*, vol. 44, no. 4, pp. 283–291, 2013.

16. K. Wang, J. Alzate, and P. K. Amiri, "Low-power non-volatile spintronic memory: STT-RAM and beyond," *Journal of Physics D: Applied Physics*, vol. 46, no. 7, p. 074003, 2013.

17. C.-C. Wang and C.-P. Kuo, "200-MHz single-ended 6T 1-kb SRAM with 0.2313 pJ energy/access using 40-nm CMOS logic process," *IEEE Transactions on Circuits and Systems II: Express Briefs*, vol. 68, no. 9, pp. 3163–3166, 2021.

18. S. S. Kumar and J. Nayak, “Effective 8T reconfigurable SRAM for data integrity and versatile in-memory computing-based AI acceleration,” *Electronics*, vol. 14, no. 13, p. 2719, 2025.

19. K. T. Malladi, B. C. Lee, F. A. Nothaft, C. Kozyrakis, K. Periyathambi, and M. Horowitz, “Towards energy-proportional datacenter memory with mobile DRAM,” *ACM SIGARCH Computer Architecture News*, vol. 40, no. 3, pp. 37–48, 2012.

20. H. Sun, L. Chen, X. Hao, C. Liu, and M. Ni, “An energy-efficient and fast scheme for hybrid storage class memory in an AIoT terminal system,” *Electronics*, vol. 9, no. 6, p. 1013, 2020.

21. L. Zhu, L. Zhu, S. Shi, D. C. Ralph, and R. A. Buhrman, “Energy-efficient ultrafast SOT-MRAMs based on low-resistivity spin hall metal $Au_{0.25}Pt_{0.75}$,” *Advanced Electronic Materials*, vol. 6, no. 2, p. 1901131, 2020.

22. E. Grimaldi, V. Krizakova, G. Sala, F. Yasin, S. Couet, G. Sankar Kar, K. Garello, and P. Gambardella, “Single-shot dynamics of spin-orbit torque and spin transfer torque switching in three-terminal magnetic tunnel junctions,” *Nature nanotechnology*, vol. 15, no. 2, pp. 111–117, 2020.

23. S. Bhatti, R. Sbiaa, A. Hirohata, H. Ohno, S. Fukami, and S. Piramanayagam, “Spintronics based random access memory: A review,” *Materials today*, vol. 20, no. 9, pp. 530–548, 2017.

24. M. Cubukcu, O. Boulle, N. Mikuszeit, C. Hamelin, T. Brächer, N. Lamard, M.-C. Cyrille, L. Buda-Prejbeanu, K. Garello, I. M. Miron, *et al.*, “Ultra-fast perpendicular spin–orbit torque MRAM,” *IEEE Transactions on Magnetics*, vol. 54, no. 4, pp. 1–4, 2018.

25. Y. Seo and K.-W. Kwon, "Area optimization techniques for high-density spin-orbit torque MRAMs," *Electronics*, vol. 10, no. 7, p. 792, 2021.

26. C. Safranski, G. Hu, J. Sun, P. Hashemi, S. Brown, L. Buzi, C. P. D'Emic, E. Edwards, E. Galligan, M. Gottwald, *et al.*, "Reliable sub-nanosecond MRAM with double spin-torque magnetic tunnel junctions," in *2022 IEEE Symposium on VLSI Technology and Circuits (VLSI Technology and Circuits)*, pp. 288–289, IEEE, 2022.

27. R. Patel, X. Guo, Q. Guo, E. Ipek, and E. G. Friedman, "Reducing switching latency and energy in STT-MRAM caches with field-assisted writing," *IEEE Transactions on Very Large Scale Integration (VLSI) Systems*, vol. 24, no. 1, pp. 129–138, 2015.

28. P. K. Amiri, Z. Zeng, P. Upadhyaya, G. Rowlands, H. Zhao, I. Krivorotov, J.-P. Wang, H. Jiang, J. Katine, J. Langer, *et al.*, "Low write-energy magnetic tunnel junctions for high-speed spin-transfer-torque MRAM," *IEEE Electron Device Letters*, vol. 32, no. 1, pp. 57–59, 2010.

29. T. Moposita, E. Garzón, A. Teman, and M. Lanuzza, "Cryo-SIMPLY: a reliable STT-MRAM-based smart material implication architecture for in-memory computing," *Nanomaterials*, vol. 15, no. 1, p. 9, 2024.

30. K. Cai, G. Talmelli, K. Fan, S. Van Beek, V. Kateel, M. Gupta, M. Monteiro, M. B. Chroud, G. Jayakumar, A. Trovato, *et al.*, "First demonstration of field-free perpendicular SOT-MRAM for ultrafast and high-density embedded memories," in *2022 International Electron Devices Meeting (IEDM)*, pp. 36–2, IEEE, 2022.

31. Y. Wu, K. Garello, W. Kim, M. Gupta, M. Perumkunnil, V. Kateel, S. Couet, R. Carpenter, S. Rao, S. Van Beek, *et al.*, "Voltage-gate-assisted spin-orbit-torque magnetic random-access memory for high-density and low-power embedded applications," *Physical Review Applied*, vol. 15, no. 6, p. 064015, 2021.

32. V. Nguyen, S. Rao, K. Wostyn, and S. Couet, "Recent progress in spin-orbit torque magnetic random-access memory," *npj Spintronics*, vol. 2, no. 1, p. 48, 2024.

33. S. Finizio, K. Zeissler, S. Wintz, S. Mayr, T. Weßels, A. J. Huxtable, G. Burnell, C. H. Marrows, and J. Raabe, "Deterministic field-free skyrmion nucleation at a nanoengineered injector device," *Nano letters*, vol. 19, no. 10, pp. 7246–7255, 2019.

34. S. Chen, J. Lourembam, P. Ho, A. K. Toh, J. Huang, X. Chen, H. K. Tan, S. L. Yap, R. J. Lim, H. R. Tan, *et al.*, "All-electrical skyrmionic magnetic tunnel junction," *Nature*, vol. 627, no. 8004, pp. 522–527, 2024.

35. J. Urrestarazu Larrannaga, N. Sisodia, R. Guedas, V. T. Pham, I. Di Manici, A. Masseboeuf, K. Garello, F. Disdier, B. Fernandez, S. Wintz, *et al.*, "Electrical detection and nucleation of a magnetic skyrmion in a magnetic tunnel junction observed via operando magnetic microscopy," *Nano Letters*, vol. 24, no. 12, pp. 3557–3565, 2024.
